# Methods Used to Assess Physical Abilities in Police Students: A Scoping Review

**DOI:** 10.3390/sports14080356

**Published:** 2026-08-18

**Authors:** Emilia Martinsson, Haris Pojskic, Anna Hafsteinsson Östenberg, Jesper Augustsson

**Affiliations:** 1 Department of Sport Science, Linnaeus University, 391 82 Kalmar, Sweden; haris.pojskic@lnu.se (H.P.); anna.hafsteinsson-ostenberg@lnu.se (A.H.Ö.); jesper.augustsson@lnu.se (J.A.); 2 Department of Criminology and Police Work, Linnaeus University, 351 95 Vaxjo, Sweden

**Keywords:** physical tests, physical fitness, police academy, police education, law enforcement, tactical populations, occupational fitness, recruits, cadets

## Abstract

Physical ability testing is an essential part of police education, as fitness assessments indicate students’ preparedness for police work. Clear documentation and reporting of test protocols and measurement properties are essential for interpreting, evaluating, and comparing the usefulness of different test batteries. Therefore, this scoping review aimed to map the fitness tests used in police education programs internationally, including reporting standards of test protocols, reliability, validity, and sex-based test specificity, thereby providing insight into the physical abilities considered important for police work. The main searches were conducted in the databases CINAHL, PubMed, SPORTDiscus, and Web of Science. The database search yielded 1323 articles, of which 92 met the inclusion criteria. An additional nine articles were included through citation and hand searching. Of the 101 studies, 95 used an average of 4–5 general fitness tests, and 29 included work-related police tests. Test protocols were generally well documented across studies. However, only a few studies included in this review assessed test–retest reliability, and only a limited number of studies, whose primary aim was to validate the tests, evaluated test validity. The most commonly used assessments were upper-body and core muscular endurance (60 s push-up, maximum pull-up, and 60 s sit-up tests), upper-body muscular strength (hand-grip test), lower-body power (standing long jump, and vertical jump tests), and aerobic capacity (20 m multistage fitness, 2400 m Cooper, and 12 min Cooper tests. Only 19 studies reported sex-specific performance standards, including differences in test execution, scoring systems, VO_2max_ estimation, or time limits. The findings indicate that tests of upper-body muscular endurance and strength, lower-body power, and aerobic capacity are among the most frequently measured physical abilities in police education programs internationally, while also highlighting the need for more standardized reporting of test protocols, reliability, and validity.

## 1. Introduction

The nature of police work is known to be both mentally and physically demanding, involving duties that sometimes require substantial physical effort and preparedness [1,2,3]. Most of the physically demanding tasks performed involve activities such as running, crawling, jumping, dragging, lifting, climbing, pushing, pulling and vaulting, as well as controlling or restraining individuals when required [4,5]. Consequently, an adequate level of physical fitness is generally considered important for a police officer to perform these tasks effectively and safely [6]. Before becoming a police officer, there are certain requirements that new candidates must achieve. First, candidates must undergo a successful selection or recruitment process before being accepted into a police education program. This process typically includes medical and psychological assessments, and physical testing. Second, police students are then required to complete both academy training and field training [7]. However, both recruitment procedures and police education programs vary substantially across countries, regions and states, with respect to their structure, duration, requirements, and intended preparation for future police work [7,8,9]. Physical ability training and testing are commonly included in police education programs to prepare students for occupational demands, such as minimizing injury risk and optimizing performance [10,11]. However, despite the widespread use of physical ability testing in police education, there is limited overview of which physical fitness assessments are used internationally and how these tests are reported in scientific literature.

Physical fitness is commonly described as the capacity to perform physical activities efficiently and effectively and is often categorized into health-related and skill-related components [12]. Health-related fitness refers to physical capacities such as muscular strength, muscular and cardiovascular endurance, flexibility, and body composition, whereas skill-related fitness encompasses attributes such as power, speed, agility, balance, coordination, and reaction time [12]. In this context, physical ability may be understood as an individual’s capacity to perform movement tasks requiring combinations of these physical attributes [13]. Within the police population, higher levels of physical fitness have been associated with improved performance in occupational tasks and a lower risk of injury [14,15]. Consequently, physical ability testing is commonly included in both police recruitment procedures and police education programs, for example as entry tests, training assessments, or graduation requirements [16]. Physical ability testing as part of police education programs typically includes both general fitness assessments (e.g., push-up, sit-up, sit-and-reach, grip strength, vertical jump, standing long jump, multistage fitness tests, and the Cooper test), as well as work-related police tests designed to simulate occupational tasks (e.g., obstacle courses, dragging tasks, and carrying tasks) [17,18,19]. While general fitness tests primarily assess underlying physical capacities, work-related police tests aim to replicate and assess task-specific abilities and skills relevant to policing duties [18,19]. Several work-related police test batteries have been developed and implemented within police populations, including the Police Officer’s Physical Ability Test (POPAT); The Physical Ability Requirement Evaluation (PARE); Officer Physical Ability Test (OPAT) [1,20]; Physical Appraisal Test (PAT); Physical Competency Test (PCT) [17,21,22]. Because these assessments are intended to evaluate an individual’s ability to perform police duties, it is important to understand how physical fitness relates to occupational performance.

Several studies have reported associations between physical fitness and performance in police occupational tasks [20,23,24]. For example, muscular strength, muscular endurance (often assessed using push-ups and sit-ups), and aerobic and anaerobic capacity have been linked to improved performance in work-simulated policing tasks [3,25]. Specific tests commonly used within police populations, such as body drag tasks, have also been shown to be associated with measures of strength and power [26,27,28,29]. In addition, other fitness-related factors including body composition, flexibility, and aerobic capacity have been reported to influence performance in work-simulated policing tasks [30,31].

Despite the established importance of physical fitness for police performance, physical test batteries used in police education vary across countries and institutions and typically include a combination of general fitness assessments and work-related police tests [25,32,33]. Consequently, the structure and content of physical ability testing differ substantially, for example with respect to whether test performance standards are sex-neutral or sex-specific. For instance, in the United States (e.g., California), police students are required to complete the same training and assessment (e.g., the Work Sample Test battery—WSTB) to graduate, reflecting “one-size-fits-all” approach based on the assumption of uniform occupational demands [33]. In contrast, sex-specific test performance standards are implemented in police education programs in various international contexts. Despite these differences, higher levels of physical fitness and better performance in occupational tasks are consistently associated with successful graduation. This variation highlights the importance of examining how physical ability testing is currently implemented in police education [8].

Given the widespread use of physical ability testing in police education, the quality of the tests themselves is also important. Physical ability tests should demonstrate adequate validity by accurately reflecting the physical demands of police work, while also being sufficiently reliable to distinguish between individuals and detect meaningful changes over time [1,4].

Previous reviews on this topic have reported a wide range of fitness assessments used within broader police populations [14,19,34,35,36]. However, they have not specifically examined the fitness tests and test batteries used within police education programs internationally, nor how these assessments are described and reported in the literature with respect to test protocols, reliability, validity, and sex-specific performance standards. Therefore, there is still a limited overview of the methods used to assess physical fitness in police education programs. Accordingly, this scoping review aimed to map the fitness tests and outcome variables used in police education programs internationally, with a particular focus on the reporting of test protocols and measurement properties, such as reliability, validity, and sex-specific performance standards.

## 2. Materials and Methods

### 2.1. Protocol and Registration

A scoping review design was used to map, collate and summarize the research-based evidence on the methods and fitness assessment variables employed in police education programs internationally. This scoping review was conducted aligned with the methodological framework developed by Arksey and O’Malley [37] consisting of the five stages: (1) identifying the research questions, (2) identifying relevant studies, (3) study selection, (4) charting the data, and (5) collating, summarizing, and reporting the results. The protocol was guided by the Joanna Briggs Institute’s (JBI) guidance [38,39] for scoping reviews, and reported according to the preferred reporting items for scoping reviews (PRISMA-ScR) [40]. The protocol was registered at the Open Science Framework (OSF) on the 2 June 2025 (10.17605/OSF.IO/U7W8E, https://osf.io/u7w8e/overview, accessed on 12 August 2026).

### 2.2. Eligibility Criteria

This scoping review included all English-language peer-reviewed original research studies published in scientific journals. There was no limitation on the publication year. Other sources, such as conference papers, proceedings, abstracts, essays, dissertations, posters, discussion articles and papers, and presentations were excluded. Other sources were excluded to refine the study selection and ensure that the review focused solely on full versions of published, peer-reviewed articles. The authors additionally decided to exclude other reviews, as well as unpublished and grey literature, from this review. The eligibility criteria for this scoping review were developed using the PCC (Participants, Concept, and Context) framework [39]. Participants included all police and law enforcement students (cadets, trainees, candidates, applicants, and recruits). In this scoping review, the terms applicant and candidate refer to individuals in the process of being accepted into a police education program (e.g., pre-academy, selection and recruitment phases, or the initial hiring process). The terms cadet, trainee, recruit, and student refer to individuals engaged in a police education program [8]. Although the studies in this review used a variety of terms to describe their participants (e.g., law enforcement recruit, police recruit, police trainee, law enforcement agency recruit, police academy cadet, police college trainee, law enforcement candidate, and law enforcement applicant), this review uses the terms police student (individual engaged in a police education program) and police candidate (individual undergoing a recruitment process). Studies that included both police or law enforcement students and police officers or law enforcement personnel were also included. Studies that included both police or law enforcement students and police officers or law enforcement personnel were also included if data for the student/candidate population were reported separately. In such cases, only the student/candidate data were extracted for analysis. Studies focusing on populations other than police or law enforcement personnel, such as military service members, emergency responders, or other tactical groups, were excluded. The concept explored in this review was any method used to assess physical ability as part of the selection or recruitment process (e.g., entry tests, initial testing prior to academy training, screening, recruitment, and hiring) or as a regular component of police education programs (e.g., baseline assessments, routine testing procedures, and graduation requirements). However, only physical ability tests were included in the analysis; exercise activities conducted as part of training programs were excluded. The context explored in this review included all studies in which physical assessments were conducted as part of, or in relation to, police education programs. This encompassed aspects such as program content, duration, location, routine assessment procedures, and standard testing methods.

### 2.3. Search Methods

A search strategy was developed by the first author (E.M.) in consultation with an academic librarian, using relevant keywords and phrases to search across multiple databases. The search strategy was developed in consultation with an experienced academic librarian but was not formally peer reviewed using the Peer Review of Electronic Search Strategies (PRESS) guidelines. An initial limited search of PubMed (U.S. National Library of Medicine (NLM), National Center for Biotechnology Information (NCBI) and SPORTDiscus (EBSCOhost) was undertaken to chart the area of the topic, and to identify relevant articles. A selection of relevant articles was used to identify commonly used keywords and phrases in the field. These keywords and phrases, identified from the titles and abstracts of the selected articles, were then used to develop the search strategy. The search strategy was developed by identifying relevant keywords, phrases, and Medical Subject Headings (MeSH) in PubMed. The final search strategy, with keywords organized by participants and concept categories, was initially applied in the PubMed database. The final search strategy was further adapted for each electronic database by incorporating its specific keywords and controlled vocabulary (i.e., subject headings and index terms). The searches were conducted in the following databases: CINAHL with Full Text (EBSCOhost), PubMed (U.S. NLM), NCBI, SPORTDiscus (EBSCOhost) and Web of Science (Clarivate). The main searches were conducted in these selected databases between 4 August 2025 and 8 August 2025, and updated on 10 April 2026, by the first author (E.M.) (See Supplementary File S1). The follow-up search was limited by date to capture newly published literature added to the databases after the main searches were conducted. A hand-search was conducted by the first author to identify any article that could have been missed during the database searches. This hand-search also included examining the reference lists and citations of all articles included in the review. This stage of the search process was conducted to identify potential articles that met the review’s eligibility criteria but were not captured in the primary electronic database search.

### 2.4. Study Selection

Identified articles from the database searches were collated and exported into EndNote 21.5 (Clarivate, Philadelphia, PA, USA), where duplicates were removed. Two reviewers (E.M., J.A.) screened all identified articles independently by title and abstract for relevance. Prior to this step, a pilot screening test was conducted to establish inter-screener reliability for the title and abstract screening phase. The pilot screening served as an initial step to ensure consistency among reviewers in applying the review’s eligibility criteria. Five randomly selected articles were screened independently by the two reviewers based on the review’s eligibility criteria, after which they met to discuss each article and reach agreement. The remaining articles were then screened independently by the two reviewers (E.M., J.A.). All selected articles were subsequently summarized and compared using a Microsoft Excel spreadsheet. The spreadsheet served as a structured tool for discussing each article identified by the two reviewers and for reaching consensus on eligibility. Any uncertainties or questions regarding the selected articles during the screening phase were discussed and verified by the other reviewer. The remaining articles were then screened in full text by the first author to determine eligibility. Prior to this step, a pilot screening test was conducted to establish inter-screener reliability for the full-text screening phase. The pilot screening was conducted to ensure consistent application of the eligibility criteria during full-text screening. Six randomly selected articles were independently screened by three reviewers (E.M., J.A., H.P.), who then met to discuss each article and reach agreement based on the eligibility criteria. Once agreement was reached, the first author proceeded with the remaining screening, and reasons for exclusions were documented. Any uncertainties or questions regarding articles during this phase were discussed with the second author, and, when necessary, consensus was reached with the involvement of a third author. This scoping review focused on how the included studies described assessments as measures of physical ability and fitness, as well as the extent to which they described, reported, and documented physical tests. The review did not examine test results or outcome measures, such as the effectiveness of interventions.

### 2.5. Data Extraction

An extraction table was developed in Microsoft Excel and used to extract relevant data from the included studies. Data extraction was primarily conducted by the first author (E.M.) and confirmed by a second author (H.P.). Prior to this phase, a pilot screening test was conducted to ensure consistency in the extraction tool. Five randomly selected studies were independently extracted by two reviewers (E.M., H.P.), who then met to discuss each study using the extraction table. The extraction phase was preceded by the selection of 5% of all included studies, which were extracted in duplicate. This step was undertaken to ensure consistency and accuracy in the extraction phase. The extracted data were either discussed or confirmed by both reviewers. The extraction table was iteratively modified throughout the process as each study was extracted. The first author (E.M.) proceeded with the extraction of all remaining studies. Data regarding publication year, country of origin, methodological approaches, and key findings relevant to the objectives and research questions of the review were extracted. Any disagreements or uncertainties encountered during the extraction phase were resolved through discussion between the authors.

### 2.6. Data Management and Analysis

Descriptive data analysis was conducted in relation to the study characteristics. Study characteristics included authors, publication year, country of origin, and study design. Data synthesized included participants’ descriptions (e.g., male and female, police candidates and police students), age, sample size, anthropometric measurements (e.g., body height, body mass and body fat percentage), and health parameters (e.g., blood pressure and heart rate). Data analysis included the number and characteristics of the physical tests used (i.e., general fitness tests and work-related police tests), the measures of physical capacity (e.g., muscular strength, muscular endurance, flexibility and agility), and the stated purpose of each test used (e.g., as part of the police education program, as part of the recruitment process, or research). The determination of how the tests were used was based on each study’s stated primary purpose for the tests. The analysis also included the number of studies examining test validity, the reporting of test–retest reliability, the completeness of test protocol reporting, and the reporting of sex-specific performance standards. The reporting of test–retest reliability was classified into three levels: (i) assessed and reported within the study; (ii) not assessed but supported by reliability evidence from previous studies; or (iii) not reported. Reliability was evaluated solely based on reporting completeness. The assessment did not consider the quality of the reported reliability. Test validity was recorded only when the primary aim of the study was to validate the test. The assessment did not consider whether the test had been validated previously. The reporting standards of the test protocols were evaluated using a 20-item reporting framework comprising four domains: (i) general test information (3 points), (ii) equipment and setup (6 points), (iii) test protocol description (8 points), and (iv) test units and conventions (3 points) (see Supplementary File S2). The overall reporting standard was classified as low (<11), medium (11–<16), or high (≥16). Studies receiving a half-point score were classified according to the predefined thresholds, with the full point value required to reach the next reporting category.

The reporting standards classification framework was developed specifically for this scoping review and was not designed to evaluate the methodological quality of assessment methods or test protocols. Instead, it was used solely to classify the level of reporting in the included studies. Sex-specific performance standards were analyzed only in studies that used general fitness tests and work-related police tests as part of a police education program (e.g., for entry screening, recruitment and selection, baseline assessments, routine testing, or graduation requirements). The analysis examined whether studies reported sex-specific performance standards.

Prior to data extraction, two authors (E.M., H.P.) independently evaluated 10 randomly selected articles and then met to compare their assessments until consensus was reached. This step was conducted to ensure consistency among reviewers in evaluating the reporting standards of the included studies. The subsequent evaluation of reporting standards was conducted by the first author (E.M.). Data was presented in tables and figures, accompanied by a summary outlining the findings in relation to the scoping review’s objectives and research questions. Modifications to the included information in the data analysis and the presentation of results were made throughout the process.

## 3. Results

**Figure 1:**
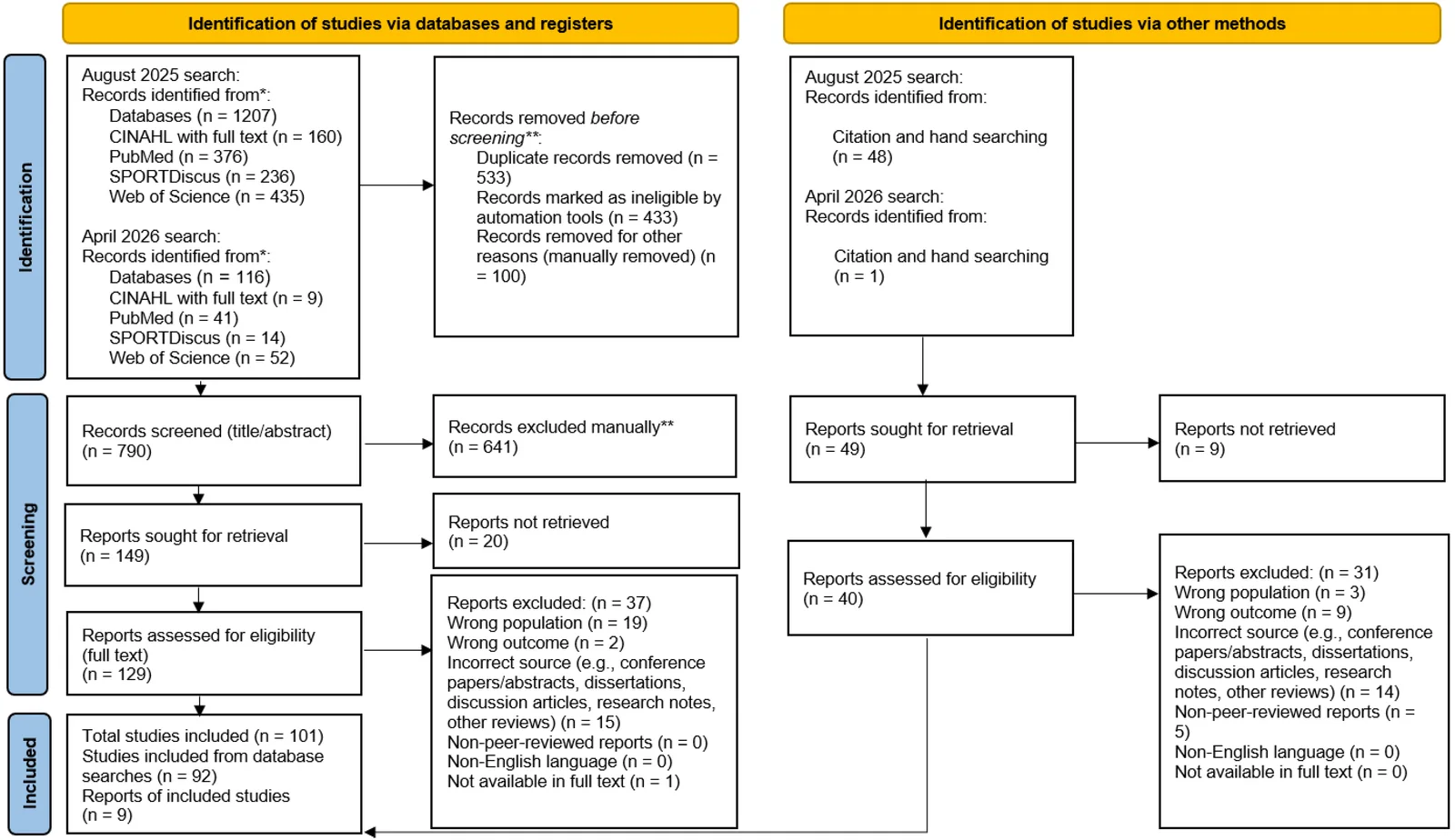
PRISMA flowchart depicting the selection process and reasons for exclusions. * The reporting of the number of records identified from each database or register searched (rather than the total number across all databases/registers). ** Indication of how many records were excluded by a human and how many were excluded by automation tools.

The search in four databases yielded a total of 1323 articles. After removing duplicates, 790 articles remained for title and abstract screening. After excluding irrelevant articles, 129 articles were retrieved in full-text versions and screened for eligibility criteria, resulting in 92 articles included in the review. An additional 49 articles were identified through screening citations of the included articles, and hand searching. Nine of these articles met the eligibility criteria, resulting in a total of 101 articles included in this scoping review (see Figure 1 for detailed selection process and reasons for exclusions).

### 3.1. Study Characteristics

The included studies (*n* = 101) were conducted across 17 countries, with the majority in the USA (~50%), Serbia (~17%), and Australia (~11%). In total, nine research designs were identified among the selected studies. Most of the included studies used a retrospective cross-sectional (~52%), a cross-sectional (~16%), an observational cohort (~9%), or retrospective longitudinal cohort (~9%) design. Only a limited number of studies utilized prospective cohort (*n* = 3) or randomized controlled trial (*n* = 3) study designs. Six studies were methodological studies.

### 3.2. Participants’ Characteristics

In the 101 studies, the total sample size was 43,907. Based on 89 studies, there were 30,599 male participants, while 82 studies reported a total of 8908 female participants. Based on 74 and 69 studies, respectively, the average age of male and female participants was similar, (25.6 and 25.5 years, respectively), with a range of 18 to 55 years. Only twelve studies reported body composition data (e.g., body fat percentage and muscle mass percentage), whereas body height and mass were reported in 74 and 81 studies, respectively. Body mass index (BMI) was reported in 29 studies. Body circumference data were reported in only 10 studies. Blood pressure was measured in two studies, while blood lactate was measured in three studies. Heart rate measurements were included in four studies. For security reasons, demographic and anthropometric data were not always disclosed in the studies.

### 3.3. Test Protocols Used to Assess Physical Abilities

Our results indicate that between four and five general fitness tests were employed across 95 studies, with a minimum of one test and a maximum of 13 tests. A variety of work-related police tests were employed across 29 studies. Among the general fitness tests, 60 s push-ups [11,16,41,42,43,44,45,46,47,48,49,50,51,52,53,54,55,56,57,58,59,60,61,62,63,64,65,66,67,68,69], maximum pull-ups [25,32,69,70,71,72,73,74,75,76,77,78,79,80], and 60 s sit-ups [11,16,41,42,43,44,45,46,47,48,49,50,51,52,53,54,55,56,57,58,59,60,61,62,63,64,65,66,67,68,69,70,71,80,81,82,83,84,85,86] were the most commonly used measures of upper-body and core muscular endurance (Table 1a). In contrast, lower-body muscular endurance was assessed in only one study [11]. Of the 95 studies included, ~62% used physical tests to assess upper-body muscular endurance, whereas ~64% assessed core muscular endurance. Upper body-strength was assessed in ~44% of the studies, with the hand grip test being the most frequently used measure [9,11,16,21,22,26,41,49,53,59,60,62,63,66,67,69,71,75,81,82,86,87,88,89,90,91,92,93,94,95,96,97]. In contrast, lower-body strength was assessed in only ~6% of the included studies. Agility and coordination were evaluated in ~36% of the selected studies, with the 75-yard pursuit run test used on 15 occasions [32,44,46,48,53,55,58,59,60,61,63,68,77,78,98]. The standing long jump test [9,27,66,69,71,72,75,76,80,81,82,86,87,89,90,92,93,95,97,99,100,101,102] and the vertical jump (jump-and-reach) test [11,21,22,27,32,45,47,49,53,55,58,59,60,63,64,68,96,98,103,104,105] were the most commonly used measures of lower-body power, while upper-body power was most frequently assessed using the 2 kg medicine ball throw test [32,48,53,55,58,59,60,63,68,77,78,103] (Table 1b). Lower-body power was assessed in ~49% of the selected studies using various physical tests, while upper-body power was assessed in ~15% of the studies. Running speed was assessed in only 12% of the studies included. Aerobic capacity was evaluated in ~89% of the selected studies. The most frequently employed assessments were the 20 m multistage fitness test [32,45,48,51,52,53,54,55,58,59,60,63,66,67,68,69,71,77,78,81,82,86,99,106,107,108,109,110,111,112,113,114,115,116,117], the 2400 m Cooper test [16,21,22,25,32,41,42,43,44,46,47,50,52,56,57,61,62,64,65,70,73,77,78,79,84,85,88,96,106,107,110,111,118], and the 12 min Cooper test [9,69,75,89,90,92,93,95,100,101,119,120]. In contrast, only ~18% of the studies included measures of anaerobic capacity. Flexibility was evaluated in ~15% of the included studies, with the sit-and-reach test being the most frequently used assessment [11,16,41,49,56,62,65,69,70,79,80,86,87,88]. In contrast, only ~3% of the studies included measures of mobility and only 5% included measures of motor educability. Notably, the number of tests reported in Table 1a,b may exceed the number of studies for a given capacity, as some studies used multiple tests to assess the same capacity.

A total of 17 different work-related police tests were identified across 29 studies. The body drag test [26,27,126,127] and the work sample test battery [25,32,33,77,78] were the most frequently used tests, representing ~14% and ~17% of the included studies, respectively (Table 2). Notably, the body drag test was also included as a part of the work sample test battery. The obstacle course for the assessment of specific abilities of police officers [90,128], the physical ability test [16,41], the physical competency test [17,21], and the physical performance evaluation [114,116] were used in two studies, respectively. Notably, the police-specific physical ability test was performed in the same way the obstacle course for the assessment of specific abilities of police officers. The characteristics of the tests provided a varied range of different tasks, with the most frequent being lifting, dragging, climbing, crawling, pushing, pulling, running, and carrying. Most of the work-related police tests were designed as obstacle courses, with stations that simulated different tasks to imitate real police work situations.

Twenty-three studies used both general fitness tests and work-related police tests. In most studies (~73%), the tests were conducted within police education (i.e., baseline assessments, routine testing procedures or graduation requirements). In ~13% of the studies, the tests were part of the selection or recruitment process (i.e., entry examinations or hiring procedures), while ~25% of the studies used these tests specifically for research purposes. In eight studies, the tests were used both within police education and for research purposes. Only three studies used the tests both within police education and as a part of the selection or recruitment process.

### 3.4. Reporting Standards of Test Protocols

The majority of the included studies (~80%) were evaluated as having a high reporting standard for the test protocols used (See Supplementary File S3). In contrast, seventeen studies were rated as having a medium reporting standard, and four were classified as having a low reporting standard.

### 3.5. Test–Retest Reliability and Validity

Among the studies employing general physical fitness tests (*n* = 95), four studies assessed and reported test–retest reliability within the studies, 28 studies reported reliability supported by previous evidence, and 63 neither assessed nor reported reliability (See Supplementary File S4). Three studies conducted test validation. Among the studies employing work-related police tests (*n* = 29), two studies assessed and reported test–retest reliability within the studies, four studies reported reliability supported by previous evidence, and 23 neither assessed nor reported reliability. One study conducted test validation and also assessed and reported test–retest reliability within the study.

### 3.6. Sex-Specific Reporting of Test Performance

Among the 84 included studies that used tests as part of police education programs (e.g., entry screening, recruitment and hiring, baseline assessments, routine testing, or graduation requirements), only 19 studies reported sex-specific standards. The reported sex-specific standards included differences in test execution, VO_2max_ estimation, scoring systems, and time limits. For general fitness tests, sex-specific variations in execution were most commonly reported for the push-up test, followed by the pull-up test [16,41,56,62,69,72]. Some studies reported procedural differences, such as pairing female participants with female testers during the push-up test, without differences in performance standards. Other studies applied longer time limits for female participants in running tests (e.g., the 2000 m run, the Harres test, the 2400 m Cooper test, and the 12 min Cooper test) [7,56,119]. Several studies used different equations to estimate VO_2max_ for female and male students or applied sex-specific scoring systems based on summed test battery scores and BMI. For example, three studies used sex-specific equations to estimate VO_2max_ from the 2400 m Cooper test [109,110,111], while one study did so based on the 20 m multistage fitness test [108]. One study required both a passing score of 11 points and a minimum event-specific score of 1 point for both sexes, with additional points awarded to students with higher BMI if their run time met or exceeded the passing threshold [96]. One study also reported that participants were required to meet time standards that varied by both sex and age across multiple tests (e.g., the 1600 m run, sit-ups, and burpees) [11]. A further study applied sex-specific performance standards using different tests for muscular endurance (pull-ups for men and sit-ups for women) and aerobic endurance (1000 m run for men and 800 m for women), based on sex-specific national norms and scoring systems [80]. Another investigation used different resistive loads for male (5%) and female students (2%) during the arm crank test [49]. For work-related police tests, four studies specified sex-specific standards, including differences in test execution and time limits. For example, one study reported different execution standards for the body drag test, where female students had the option to drag the dummy rather than carry it [129]. Additionally, two studies applied age- and sex-specific completion time standards for the physical competency test [17,21].

## 4. Discussion

The aim of this scoping review was to provide an overview of the evidence on the methods and fitness tests used in police education programs internationally. To the best of our knowledge, this is the first study to systematically map and collate the methods used to assess physical abilities with a specific focus on police students and candidates, with particular attention to the reporting and documentation of test protocols in police education programs. Most studies in this review employed a combination of general fitness tests and work-related police tests as part of their test batteries. In addition, the work-related police tests consisted of diverse tasks, primarily structured as obstacle courses simulating real police work. Upper-body muscular endurance and strength, lower-body power, and aerobic capacity were the most commonly assessed physical abilities. Although the reporting of test protocols was classified as high in most studies, relatively few assessed and reported test–retest reliability, and only a limited number reported reliability values from previous research. A similar pattern was observed regarding the validity of the test protocols included, as only a small number of studies had conducted or reported test validation. Likewise, reporting of sex-specific differences in test performance standards was limited, with only a few studies providing sex-specific performance criteria.

### 4.1. Physical Ability Assessments and Tests in Police Education Programs

Internationally, the most employed physical fitness assessments were: (i) 60 s push-ups and 60 s sit-ups for upper-body and core muscular endurance; (ii) grip strength for upper-body strength; (iii) the vertical jump (jump-and-reach) and standing long jump for lower-body power; and (iv) the 20 m multistage fitness test, Cooper test (2400 m and 12 min), for aerobic capacity. Most of the tests included in this review were field-based, requiring minimal, less expensive equipment and allowing many students to be tested at the same time, making them suitable for police education. Several of these tests have also been highlighted in the previous literature as commonly used within police education programs [17,18,19]. For example, push-up and sit-up tests have been shown to predict occupational performance in policing duties [3]. In this review, aerobic capacity was identified as the most frequently employed measure of physical ability, which is consistent with its established importance for occupational performance [25,30,31]. Furthermore, muscular endurance, upper-body strength, and lower-body power were consistently identified as relevant for effective police performance and have been shown to improve performance in work-related police tests [25,26,27,28,29]. Despite the recognized relevance of anaerobic capacity and lower-body strength for tasks such as body drag [25,26,27,28,29], these abilities were less frequently assessed in the included studies. Regarding agility and coordination, the 75-yard pursuit run was the most commonly employed test, while other assessments of these abilities were used less frequently. Body composition, flexibility, and mobility were relatively underrepresented as assessment measures. This may indicate that certain relevant physical capacities are under-assessed in police education programs, despite their potential contribution to performance in work-related tasks [30,31]. One possible explanation is that assessments of body composition often require specialized equipment, such as a dual-energy X-ray absorptiometry (DXA) scanner or bioelectrical impedance analyzer, which may limit their practical implementation.

Regarding the work-related police tests, the body drag test and the work sample test battery were the most commonly used. The second most frequently used tests were the obstacle course for the assessment of specific abilities for police officers, the physical ability test, the physical competency test, and the physical performance evaluation. These tests typically consist of tasks such as lifting, dragging, climbing, crawling, pushing, pulling, running, and carrying, often performed within an obstacle course. Previous research has emphasized that these tasks represent some of the most frequently physically demanding tasks among police officers [4,5]. Consequently, many of these tasks are included in test batteries within police education programs [16]. Test requirements vary internationally; for example, time standards for the body drag test may differ between countries and states. For example, in California (USA), the body drag test must be completed within 28 s over 9.75 m, and successful competition of the work sample test battery is required for graduation [25,26,32,33]. In New Zealand, for example, the body drag test included in the physical competency test requires students to lift and drag a 75 kg dummy over 7.5 m [17].

Our findings indicate that on average four to five general fitness tests were employed across 95 studies, and 29 studies included work-related police tests. Of these, 23 studies employed both general fitness and work-related police tests within their test batteries. These findings emphasize the importance of incorporating a variety of physical ability assessments in police education programs for several reasons. Higher levels of fitness are associated with: (i) improved performance in occupational tasks [20,23,24]; (ii) a reduced risk of injury [14,15]; (iii) improved preparedness for future police work [10,11]; and (iv) an increased likelihood of successful graduation [8]. From an international perspective, our findings suggest that test batteries used within police education programs typically comprise multiple assessment tests to evaluate different aspects of physical ability.

Most tests identified in this review were field-based and are generally easy to administer. While these tests offer practical advantages (e.g., low cost and feasibility in large cohorts), their predominance may also reflect the limited adoption of more advanced and contemporary methods for assessing physical capacity. Given the substantial duration and resource investment associated with police education programs, there appears to be scope for integrating more comprehensive and sensitive testing methods that better capture the physical demands of modern police work. When designing test batteries for police students, priority should therefore be given to tests that are valid, sensitive, and relevant to the occupational demands of future police work, rather than prioritizing practical feasibility alone.

### 4.2. Evaluation and Documentation of Tests and Test Protocols

Although the majority of the studies included in this review were classified as having a high reporting standard for test protocols, approximately 21% were rated as having low to medium reporting standards. Detailed reporting of test protocols (e.g., setup description, preparation and warm-up, positioning and alignment, rest intervals, safety measures, and deviations from standard protocols) is crucial. Insufficient reporting reduces the ability to draw meaningful conclusions regarding the usefulness of tests in police education programs and limits their repeatability in a scientific context. This may be particularly important for work-related police tests, which often consist of multiple tasks performed within complex obstacle courses. Notably, most studies that included work-related police tests in this review provided illustrations of the test courses. However, only a small number of studies evaluated and reported test–retest reliability, and the majority did not report reliability values from previous research. Consequently, the reliability of the test protocols employed cannot be adequately judged. Similarly, few studies conducted validation of the tests used. However, these findings should be interpreted with caution, as the present review assessed whether reliability information was reported, but did not evaluate the quality of the reported estimates. Similarly, validity was recorded only when test validation was a primary aim of the included study. Thus, the absence of reported reliability or validity should not be interpreted as evidence that a test is unreliable or invalid, as such evidence may have been established in previous research. Given that work-related police tests should simulate police-specific tasks [18,19], the limited evaluation and reporting of reliability and validation may compromise their interpretability and practical usefulness. Regarding the general fitness tests (e.g., push-up, sit-up, vertical jump, etc.), the present review suggests that reporting of test–retest reliability is often insufficient despite their widespread use [34,35].

Although approximately 80% of the studies achieved a high overall reporting standard, the general information domain was at or near maximum score for almost all studies and therefore contributed little to differentiating between studies. Reporting deficiencies were primarily related to equipment and setup and test protocol description, indicating that these domains represent the main areas for improvement.

### 4.3. Sex-Specific Differences in Test Performance Standards

The majority of the included studies in this review did not specify whether test performance standards were sex-specific or sex-neutral. However, despite the limited reporting of such procedures, sex-specific test variations, criteria, or scoring standards may still have been implemented in practice within certain police education programs or jurisdictions. For example, Lockie et al. [33] stated that the same requirements for both male and female police students may be applied in specific test batteries. However, the findings of this review reveal considerable variation in sex-specific performance standards across countries. More specifically, studies reporting sex-specific performance standards in police education programs were conducted in the USA, Australia, Germany, New Zealand, the Netherlands, Sweden, Serbia, Norway, Denmark, Portugal, and China. Notably, some of the included studies enrolled only female or only male participants; therefore, reporting test performance differences between sexes may not be relevant in those cases.

Although this review systematically evaluated reporting standards, as well as the reporting of reliability, validity, and sex-specific test performance standards, it was not designed to determine which testing models or performance standards are most appropriate for police education programs. Nevertheless, the findings highlight the importance of detailed and standardized reporting of test protocols.

### 4.4. Strengths and Limitations

A major strength of this scoping review is its systematic approach, guided by established scoping review frameworks. The search strategy identified a large number of relevant studies, enabling a comprehensive overview of the available evidence related to the topic. The review also identified important gaps in the literature that may help guide future research and the development of more standardized testing and reporting practices in police education. However, it was limited to English-language, peer-reviewed studies and excluded grey literature. Although the search strategy was developed in collaboration with an academic librarian, optimized for each database, and supplemented by citation tracking and hand searching, it was not formally peer-reviewed using the PRESS guidelines. Consequently, some relevant studies using alternative terminology may not have been identified. Additionally, a limitation of this review is that the selected search terms may not have captured all relevant studies on the topic, potentially resulting in the omission of some available evidence. Restricting the review to English-language, peer-reviewed publications may have excluded some relevant sources. However, this approach ensured that the synthesis was based on studies meeting established scientific and reporting standards, which was particularly important given the review’s focus on test protocols, reliability, validity, and reporting quality. It should be noted that the classification of reliability, validity, and reporting standards was based on a non-validated framework developed specifically for this review. This review assessed only the reporting of reliability and validity within the included studies. Therefore, the findings should not be interpreted as evidence of the actual reliability or validity of the physical tests used. Reliability classifications reflected the extent to which reliability was reported, while validity classifications were based solely on whether test validation was the primary aim of the study. As a result, reliable and valid tests may have been used in studies that did not explicitly report the properties. The reporting-scoring framework was intended as a descriptive classification of reporting completeness rather than a formal assessment of methodological quality. Furthermore, one reviewer was primarily responsible for assessing the reporting standards of most included studies following an initial screening exercise involving two reviewers. As one reviewer primarily conducted the full-text screening and extracted and evaluated most of the included studies, the possibility of reviewer bias cannot be excluded. However, although one reviewer conducted the majority of the screening, data extraction, and assessment procedures, at least one additional reviewer was available throughout these stages to discuss uncertainties and support decision-making when required.

As approximately half of the studies included were conducted in the USA, where the WSTB is commonly used, the available evidence was geographically dominated by specific geographical and research contexts. This should be considered when interpreting the relative frequency of different physical testing methods reported in the literature. Nevertheless, the large number of included studies and the broad international representation provide a comprehensive overview of current testing methods in police education.

## 5. Conclusions

Physical tests used in police education programs internationally encompass a wide range of fitness components and job-specific tasks. Tests of upper-body muscular endurance and strength, lower-body power, and aerobic capacity were the most frequently used assessments across the included studies. These findings provide a useful overview for police educators and practitioners of the physical fitness assessments currently used in police education programs internationally. The review also identified important gaps in the current literature. Although most studies were classified as having a high reporting standard for test protocols, several studies still provided only moderate or limited methodological detail. Moreover, only a few studies assessed test–retest reliability or conducted test validation, both for general fitness tests and work-related police tests. Among studies of general fitness tests, only four assessed test–retest reliability, and only three evaluated test validity. Among studies of work-related police tests, only two assessed test–retest reliability, while only one evaluated test validity. These findings highlight important limitations in the current literature, particularly regarding the evaluation and reporting of reliability and validity, and emphasize the need for more standardized and transparent reporting of physical testing procedures, including test protocols and measurement properties, in police education research.

## Figures and Tables

**Table 1:** (**a**) Distribution of evaluated physical abilities and utilized tests across the selected studies (*n* = 95). (**b**) Distribution of evaluated physical abilities and utilized tests across the selected studies (*n* = 95).

Physical Capacity (PC)	General Physical Fitness Tests	A Number and (%) of Studies Used a Respective Test		A Number and (%) of Studies That Assessed Respective (PC)
(**a**)				
**Muscular** **endurance** **(upper-body)**	Push-up (10 s) [9,75,92,95,100,101]	6	(6)	59 (62)
	Push-up (60 s) [11,16,41,42,43,44,45,46,47,48,49,50,51,52,53,54,55,56,57,58,59,60,61,62,63,64,65,66,67,68,69]	31	(33)	
	Push-up (120 s) [25,32,73,77,78,84,85,102,121]	9	(9)	
	Push-up (maximum reps) [21,22,70,71,74,79,96]	7	(7)	
	Hand-release push-up (30 s) [99]	1	(1)	
	Hand-release push-up (120 s) [121]	1	(1)	
	Bent arm hang [87]	1	(1)	
	Arm ergometry (60 s) [44,46,52,53,60,61,63]	7	(7)	
	Mountain Climbers (120 s) [25,32,73,77,78]	5	(5)	
	Pull-up (10 reps) [75]	1	(1)	
	Pull-up (60 s) [11]	1	(1)	
	Pull-up (maximum reps) [25,32,69,70,71,72,73,74,75,76,77,78,79,80]	14	(15)	
	Pull-up slope [119]	1	(1)	
**Muscular** **endurance** **(lower-body)**	Squats (60 s) [11]	1	(1)	1 (1)
	Burpees (60 s) [11]	1	(1)	
**Muscular** **endurance** **(core)**	Sit-up (30 s) [9,75,83,87,89,90,92,93,95,99,100,101]	12	(13)	61 (64)
	Sit-up (60 s) [11,16,41,42,43,44,45,46,47,48,49,50,51,52,53,54,55,56,57,58,59,60,61,62,63,64,65,66,67,68,69,70,71,80,81,82,83,84,85,86]	40	(42)	
	Sit-up (120 s) [25,32,73,77,78,83,102,121]	8	(8)	
	Hands behind head sit-up (120 s) [121]	1	(1)	
	Sit-up (maximum reps) [79]	1	(1)	
	Plank (maximum reps) [11]	1	(1)	
	Plank (180 s) [99]	1	(1)	
**Muscular strength** **(upper-body)**	Bench press (1 RM) [11,47,49,64,70,72,74,79,84,85,90]	11	(12)	42 (44)
	Bench press (10 reps) [76]	1	(1)	
	Grip strength [9,11,16,21,22,26,41,49,53,59,60,62,63,66,67,69,71,75,81,82,86,87,88,89,90,91,92,93,94,95,96,97]	32	(34)	
	Index finger strength (pulp-2 pinch) [91]	1	(1)	
	Inverted row pull-up (maximum reps) [72,76]	2	(2)	
**Muscular strength** **(lower-body)**	Isometric deadlift (maximum reps) [75,89,100]	3	(3)	6 (6)
	Back and lumbar isometric strength (maximum reps) [69]	1	(1)	
	Leg/back isometric strength (dynamometer) [26,67]	2	(2)	
	Half-squat (1RM) [75]	1	(1)	
**Agility and** **Coordination**	Harres test [7,66]	2	(2)	34 (36)
	L-run test [66]	1	(1)	
	Slalom test [69,71,86]	3	(3)	
	T-test [43,49,97]	3	(3)	
	Box Boomerang Test [119]	1	(1)	
	75-yard pursuit run [32,44,46,48,53,55,58,59,60,61,63,68,77,78,98]	15	(16)	
	Illinois agility test [88,90,99,118,122,123,124]	7	(7)	
	Linear sprint (20 m) [124]	1	(1)	
	68.8-m agility run [52]	1	(1)	
	Visuomotor reaction time [97]	1	(1)	
	Shuttle run 10 × 5 m [87]	1	(1)	
	Shuttle run 4 × 20 m [119]	1	(1)	
	Touch plates [87,99]	2	(2)	
(**b**)				
**Power** **(upper-body)**	Medicine ball throw (2 kg) [32,48,53,55,58,59,60,63,68,77,78,103]	12	(13)	14 (15)
	Overhead medicine ball throw [102]	1	(1)	
	Medicine ball put (9 kg) [97]	1	(1)	
**Power** **(lower-body)**	Standing long jump [9,27,66,69,71,72,75,76,80,81,82,86,87,89,90,92,93,95,97,99,100,101,102]	23	(24)	47 (49)
	Vertical jump (Abalakow test) [9,92,95,100]	4	(4)	
	Vertical jump (jump and reach) [11,21,22,27,32,45,47,49,53,55,58,59,60,63,64,68,96,98,103,104,105]	21	(22)	
	Vertical jump (CMJ) [51,67,88]	3	(3)	
	Vertical jump (SJ) [88]	1	(1)	
**Aerobic capacity**	20-m Multistage Fitness Test [32,45,48,51,52,53,54,55,58,59,60,63,66,67,68,69,71,77,78,81,82,86,99,106,107,108,109,110,111,112,113,114,115,116,117]	36	(38)	85 (89)
	800 m shuttle run [49]	1	(1)	
	2000 m run [7]	1	(1)	
	12-min Cooper test [9,69,75,89,90,92,93,95,100,101,119,120]	12	(13)	
	2400 m Cooper run test [16,21,22,25,32,41,42,43,44,46,47,50,52,56,57,61,62,64,65,70,73,77,78,79,84,85,88,96,106,107,110,111,118]	33	(35)	
	800 m run test [80]	1	(1)	
	1000 m run test [80,102]	2	(2)	
	1600 m run test [11]	1	(1)	
	3000 m run test [72,119]	2	(2)	
	Direct VO_2_ max test on treadmill [113,125]	2	(2)	
	30-15 IFT [112,115]	2	(2)	
	2000 m row ergometer [76]	1	(1)	
**Anaerobic capacity**	Arm crank test (30 s) [49]	1	(1)	17 (18)
	201 m run [25,32,73,77,78]	5	(5)	
	300 m run [47,64,84,85,88]	5	(5)	
	600 m run [97]	1	(1)	
	Wingate test (30 s) [11,49]	2	(2)	
	300-yard shuttle run [90,118,122,124]	4	(4)	
	500 m rowing [11]	1	(1)	
**Running speed**	10 m sprint [90]	1	(1)	11 (12)
	10 m sprint acceleration [90,122]	2	(2)	
	30 m sprint [69,71,86,88]	4	(4)	
	40-yard sprint [49]	1	(1)	
	50 m sprint [80,99,102]	3	(3)	
	60 m sprint [69]	1	(1)	
	100 m sprint [119]	1	(1)	
**Flexibility**	Sit-and-reach [11,16,41,49,56,62,65,69,70,79,80,86,87,88]	14	(15)	14 (15)
	Hamstring, iliopsoas, multiple joint tests and shoulder joint [66]	1	(1)	
**Mobility**	The functional movement screening [123]	1	(1)	3 (3)
	Lumbar mobility [99]	1	(1)	
	Overhead squat (3 reps) [53]	1	(1)	
	Shoulder taps (screening test for postural instability) [53]	1	(1)	
**Motor educability**	Contraction and stretching [92,100]	2	(2)	5 (5)
	Whole-body contraction and extension test [9,101]	2	(2)	
	Motor educability test [95]	1	(1)	

Legend: s = seconds; reps = repetitions; 1RM = One repetition of max. CMJ = Counter movement jump; SJ = Squat jump; VO_2max_ = Maximum oxygen uptake capacity; IFT = Intermittent Fitness Test.

**Table 2:** Distribution of evaluated work-related police tests across the selected studies (*n* = 29).

Work-Related Police Tests (WRPTs)	Characteristics	Number and Percentage (%) of Studies Assessing the Respective WRPT (*n* = 29)
**The Body Drag Test (74.84 kg)** [26,27,126,127]	The task consists of lifting and dragging a dummy doll over a 9.75-m distance and placing it safely on the ground [26,27,126,127]. A 28-s time limit is required for passing the test [26,126,127]	4 (14)
**Complex Physical Task (obstacle parkour)** [119]	The task consists of three rounds and includes rope climbing, two wall rungs, a height obstacle, slalom, a longitudinal bar, a judo roll, a mat run, a box wall, a mat tunnel, a gymnastic horse, and a trestle.	1 (3)
**The Obstacle Course for the Assessment of Specific Abilities of Police Officers** [90,128]	The obstacle course (25 × 15 m of range) consists of sprints, stopping, taking cover and reaching for a firearm, holding gun in firing position and leave the cover, crawling, stopping, taking cover, change magazine and put the firearm to duty belt, crossing over an obstacle, crawling under an obstacle, punches and kicks, climbing and cross on a balance beam, leaping with a forward roll, hitting a punching bag with a baton, defending, controlling and handcuffing, sprints with a change of direction, lifting, dragging and/or carrying a dummy doll, placing a dummy doll, and running.	2 (7)
**Job-Related Task Assessment** [62]	The task includes running, crawling under an enclosure, negotiating hurdles, climbing over a ladder structure, dragging a dummy, balancing on a beam, passing through a simulated window, running up and down stairs, controlling a resisting subject, pushing and pulling a sled (twice), performing battle rope exercises, and completing an arrest procedure.	1 (3)
**Occupational Task Performance Assessments** [94]	The task comprises three components: simulation (basic defensive tactics), tactical options assessment (response to given scenarios), and marksmanship (engagement of a standard Z-4 police target using a Glock self-loading pistol).	1 (3)
**Occupational Physical Ability Test** [85]	The obstacle course consists of running and jumping, scaling and descending a barrier, crawling under obstacles, straddling a heavy bag, and performing lateral rolls, push-ups, turns, step-ups, a victim rescue, and mannequin dragging.	1 (3)
**Physical Ability Test** [16,41]	The task consists of exiting a vehicle and retrieving items from the trunk, running to an obstacle course, performing wall climbing, hurdling, navigating between pylons, crawling under a barrier, sprinting, lifting and dragging a dummy, completing the obstacle course in reverse, running back, firing and replacing the weapon in the trunk, and re-entering the vehicle.	2 (7)
**Physical Ability Test** [125]	The circuit comprises 14 tasks, including a culvert, barrier, a window, crowd navigation, stairs, a horizontal jump, side steps, a tunnel, a fence, a trash can obstacle, a balance walk, and target engagement.	1 (3)
**The Work Performance Test** [70]	The obstacle course consists of running, jumping, climbing over obstacles, identifying suspects, dragging victims, and engaging in firearm handling and controlled firing.	1 (3)
**Police Physical Tasks** [71]	The task consists of fence jumping (chasing a suspect over an obstacle), victim dragging (transporting a victim), and suspect arrest (e.g., handcuffing).	1 (3)
**Police Specific Physical Ability Test** [129]	The obstacle course consists of sprinting and stopping, taking cover and reaching a firearm, holding gun in a firing position and leaving cover, crawling under a rope, executing a magazine change, and returning the firearm to the duty belt, crossing over and crawling under obstacles, punching and kicking, climbing, balancing on a beam, rolling, hitting a punch bag with a baton and putting the baton back into duty belt, defending, controlling and handcuffing, sprinting with directional changes, carrying or dragging a dummy, placing it on the ground, and running.	1 (3)
**Physical Competence Test** [11]	The task consists of moving over a vaulting box, moving over Swedish gymnastics benches, pushing and pulling a 200 kg handcart over 6 m, and moving three 5 kg medicine balls.	1 (3)
**Physical Competency Test** [17,21]	The 400 m obstacle course consists of 10 tasks, including trailer pushing, wheel assembly carrying, running, beam balancing, jumping, vaulting, agility running, window and wall climbing, lifting and dragging a dummy, fence climbing, and sprinting.	2 (7)
**Physical Performance Evaluation(s)** [114,116]	The obstacle course consists of running and sprinting, balancing on a beam, climbing over a fence and a brick wall, jumping, hurdling, climbing, and lifting and carrying a sandbag.	2 (7)
**Scenario and shooting test** [91]	The task consists of step-ups, jumping jacks, sprawls, strikes against a heavy bag, knee strikes against a heavy bag, dragging a sled, carrying a sandbag, fighting, and dragging a dummy doll. After completing the Scenario a shooting test is performed.	1 (3)
**Standardized Physical Abilities Test** [97]	The task consists of 11 components and is performed as a continuous circuit of three laps, totaling 350 m. It is completed while wearing protective equipment weighing 6.8 kg, with a time limit of 5 min and 22 s.	1 (3)
**Use-of-Force and Arrest Simulation Test** [76]	The test comprises four elements, including use of force, standing and lying down on the ground, baton use, defense against a pointed weapon, and patrol collaboration. The total duration of the test is 90 min.	1 (3)
**Work Sample Test Battery** [25,32,33,77,78]	The task comprises a 90.53 m obstacle course, lifting and dragging a 74.84 kg dummy, climbing a 1.83 m chain-link fence, scaling a 1.83 m solid wall, and performing a 457.2 m run. A minimum score of 384 points is required for graduation.	5 (17)

## Data Availability

The raw data supporting the conclusions of this article will be made available by the authors on request.

## Supplementary Materials

The following supporting information can be downloaded at: https://www.mdpi.com/article/10.3390/sports14080356/s1. Supplementary File S1. Search strategy. Supplementary File S2. Reporting-scoring framework used to assess the completeness of test protocol reporting. Supplementary File S3. Reporting standards assessment of the included studies. Supplementary File S4. Reporting of reliability and validity of the studies included.
