# Supplementary material for: Methods Used to Assess Physical Abilities in Police Students: A Scoping Review
Source: Sports (Basel). 2026 Aug 18;14(8):356. doi: 10.3390/sports14080356 (PMC13517574; doi:10.3390/sports14080356)
Supplement: Supplementary file 1 [file sports-14-00356-s001.zip › Supplementary File S1.pdf]

Supplementary File S1. Search strategy

Source	Date of search	Search strategy (keywords, Boolean operators & Subject Headings	Search limits or filters	Results found	Comments
PubMed	2025-08-04	(“Cardiorespiratory fitness”[tiab] OR "Exercise Test"[Mesh] OR “exercise test*”[tiab] OR “fitness assessment*”[tiab] OR “fitness test*”[tiab] OR “functional performanc*”[tiab] OR "Hand Strength"[Mesh] OR “hand strength”[tiab] OR “physical abilit*”[tiab] OR “physical capacit*”[tiab] OR "Physical Endurance"[Mesh] OR “physical endurance”[tiab] OR "Physical Fitness"[Mesh] OR “physical fitness”[tiab] OR “physical performanc*”[tiab] OR “physical test*”[tiab] OR "Reference Values"[Mesh] OR “reference value*”[tiab] OR “work sample test batter*”[tiab]) AND ("Law Enforcement"[Mesh] OR “law enforcement*”[tiab] OR "Police"[Mesh] OR Police[tiab])	English	376	Exported to EndNote
SPORTDiscus	2025-08-07	((TI “aerobic fitness” OR SU “aerobic fitness” OR AB “aerobic fitness”) OR (TI “cardiorespiratory fitness” OR SU “cardiorespiratory fitness” OR AB “cardiorespiratory fitness”) OR (TI “exercise test”* OR SU “exercise test”* OR AB “exercise test”*) OR (TI “fitness assessment*” OR SU “fitness assessment*” OR AB “fitness assessment*”) OR (TI “fitness test*” OR SU “fitness test*” OR AB “fitness test”*) OR (TI “functional performanc*” OR SU “functional performanc*” OR AB “functional performanc*”) OR (TI “functional performance test*” OR SU “functional performance test*” OR AB “functional performance test*”) OR (TI “grip strength” OR SU “grip strength” OR AB “grip strength”) OR (TI “physical abilit*” OR SU “physical abilit*” OR AB “physical abilit*”) OR (TI “physical capacit*” OR SU “physical capacit*” OR AB “physical capacit*”) OR (TI “physical endurance” OR SU “physical endurance” OR AB “physical endurance”) OR (TI “physical fitness” OR SU “physical fitness” OR AB “physical fitness”) OR (TI “physical fitness test*” OR SU “physical fitness test*” OR AB “physical fitness test*”) OR (TI “physical functional performanc*” OR SU “physical functional performanc*” OR AB “physical functional performanc*”) OR (TI “physical performanc*” OR SU “physical performanc*” OR AB “physical	English; Peer Reviewed	236	Exported to EndNote

		performanc*”) OR (TI “physical performance test*” OR SU “physical performance test*” OR AB “physical performance test*”) OR (TI “physical test*” OR SU “physical test*” OR AB “physical test*”) OR (TI “reference value*” OR SU “reference value*” OR AB “reference value*”) OR (TI “work sample test batter*” OR SU “work sample test batter*” OR AB “work sample test batter*”) OR (DE "ABILITY") OR (DE "AEROBIC capacity") OR (DE "ANAEROBIC capacity") OR (DE "CARDIOVASCULAR fitness") OR (DE "EXERCISE tests") OR (DE "FLEXIBILITY testing (Physiology)") OR (DE "GRIP strength") OR (DE "JUMP & reach tests") OR (DE “MUSCLE strength”) OR (DE "MUSCLE strength measurement") OR (DE "MUSCLE strength testing") OR (DE "PHYSICAL fitness") OR (DE "PHYSICAL fitness testing") OR (DE "VERTICAL jump")) AND ((TI “law enforcement*” OR SU “law enforcement*” OR AB “law enforcement*”) OR (TI police OR SU police OR AB police))			
Web of Science	2025-08-04	(“Aerobic fitness” OR “cardiorespiratory fitness” OR “exercise test*” OR “fitness assessment*” OR “fitness test*” OR “functional performanc*” OR “functional performance test*” OR “grip strength” OR “physical abilit*” OR “physical capacit*” OR “physical endurance” OR “physical fitness” OR “physical fitness test*” OR “physical functional performanc*” OR “physical performanc*” OR “physical performance test*” OR “physical test*” OR “reference value*” OR “work sample test batter*”) AND (“Law enforcement*” OR police) (Topic)	English	435	Exported to EndNote
CINAHL (with Full Text)	2025-08-08	((TI “aerobic fitness” OR SU “aerobic fitness” OR AB “aerobic fitness”) OR (TI “cardiorespiratory fitness” OR SU “cardiorespiratory fitness” OR AB “cardiorespiratory fitness”) OR (TI “exercise test*” OR SU “exercise test*” OR AB “exercise test*”) OR (TI “fitness assessment*” OR SU “fitness assessment*” OR AB “fitness assessment*”) OR (TI “fitness test*” OR SU “fitness test*” OR AB “fitness test*”) OR (TI “functional performanc*” OR SU “functional performanc*” OR AB “functional performanc*”) OR (TI “functional performance test*” OR SU “functional performance test*” OR AB “functional performance test*”) OR (TI “grip strength” OR SU “grip strength” OR AB “grip strength”) OR	English; Peer Reviewed	160	Exported to EndNote

		(TI “physical abilit*” OR SU “physical abilit*” OR AB “physical abilit*”) OR (TI “physical capacit*” OR SU “physical capacit*” OR AB “physical capacit*”) OR (TI “physical endurance” OR SU “physical endurance” OR AB “physical endurance”) OR (TI “physical fitness” OR SU “physical fitness” OR AB “physical fitness”) OR (TI “physical fitness test*” OR SU “physical fitness test*” OR AB “physical fitness test*”) OR (TI “physical functional performanc*” OR SU “physical functional performanc*” OR AB “physical functional performanc*”) OR (TI “physical performanc*” OR SU “physical performanc*” OR AB “physical performanc*”) OR (TI “physical performance test*” OR SU “physical performance test*” OR AB “physical performance test*”) OR (TI “physical test*” OR SU “physical test*” OR AB “physical test*”) OR (TI “reference value*” OR SU “reference value*” OR AB “reference value*”) OR (TI “work sample test batter*” OR SU “work sample test batter*” OR AB “work sample test batter*”) OR (MH "Agility") OR (MH "Aerobic Capacity") OR (MH "Evaluation") OR (MH "Exercise Test+") OR (MH "Muscle Strength+") OR (MH "Physical Endurance+") OR (MH "Physical Fitness+") OR (MH "Physical Performance") OR (MH "Reference Values")) AND ((TI “law enforcement*” OR SU “law enforcement*” OR AB “law enforcement*”) OR (TI police OR SU police OR AB police) OR (MH "Police"))			
Citation searching and hand searching				48	
				Total	1255

Source	Date of search	Search strategy (keywords, Boolean operators & Subject Headings	Search limits or filters	Results found	Comments
PubMed	2026-04-10	(“Cardiorespiratory fitness”[tiab] OR "Exercise Test"[Mesh] OR “exercise test*”[tiab] OR “fitness assessment*”[tiab] OR “fitness test*”[tiab] OR “functional performanc*”[tiab] OR "Hand Strength"[Mesh] OR “hand strength”[tiab] OR “physical abilit*”[tiab] OR “physical capacit*”[tiab] OR "Physical Endurance"[Mesh] OR “physical endurance”[tiab] OR "Physical Fitness"[Mesh] OR “physical fitness”[tiab] OR “physical performanc*”[tiab] OR “physical test*”[tiab] OR "Reference Values"[Mesh] OR “reference value*”[tiab] OR “work sample test batter*”[tiab]) AND ("Law Enforcement"[Mesh] OR “law enforcement*”[tiab] OR "Police"[Mesh] OR Police[tiab])	English; Year 2025-2026	41	Updated search; Exported to EndNote
SPORTDiscus	2026-04-10	((TI “aerobic fitness” OR SU “aerobic fitness” OR AB “aerobic fitness”) OR (TI “cardiorespiratory fitness” OR SU “cardiorespiratory fitness” OR AB “cardiorespiratory fitness”) OR (TI “exercise test”* OR SU “exercise test”* OR AB “exercise test”*) OR (TI “fitness assessment*” OR SU “fitness assessment*” OR AB “fitness assessment*”) OR (TI “fitness test*” OR SU “fitness test*” OR AB “fitness test”*) OR (TI “functional performanc*” OR SU “functional performanc*” OR AB “functional performanc*”) OR (TI “functional performance test*” OR SU “functional performance test*” OR AB “functional performance test*”) OR (TI “grip strength” OR SU “grip strength” OR AB “grip strength”) OR (TI “physical abilit*” OR SU “physical abilit*” OR AB “physical abilit*”) OR (TI “physical capacit*” OR SU “physical capacit*” OR AB “physical capacit*”) OR (TI “physical endurance” OR SU “physical endurance” OR AB “physical endurance”) OR (TI “physical fitness” OR SU “physical fitness” OR AB “physical fitness”) OR (TI “physical fitness test*” OR SU “physical fitness test*” OR AB “physical fitness test*”) OR (TI “physical functional performanc*” OR SU “physical functional performanc*” OR AB “physical functional performanc*”) OR (TI “physical performanc*” OR SU “physical performanc*” OR AB “physical performanc*”) OR (TI “physical performance test*” OR SU “physical performance test*” OR AB “physical performance test*”) OR (TI “physical test*” OR SU “physical test*” OR AB “physical test*”) OR (TI “reference value*” OR SU	English; Peer Reviewed; Date range (08/2025-04/2026)	14	Updated search, Exported to EndNote

		“reference value*” OR AB “reference value*”) OR (TI “work sample test batter*” OR SU “work sample test batter*” OR AB “work sample test batter*”) OR (DE "ABILITY") OR (DE "AEROBIC capacity") OR (DE "ANAEROBIC capacity") OR (DE "CARDIOVASCULAR fitness") OR (DE "EXERCISE tests") OR (DE "FLEXIBILITY testing (Physiology)") OR (DE "GRIP strength") OR (DE "JUMP & reach tests") OR (DE “MUSCLE strength”) OR (DE "MUSCLE strength measurement") OR (DE "MUSCLE strength testing") OR (DE "PHYSICAL fitness") OR (DE "PHYSICAL fitness testing") OR (DE "VERTICAL jump")) AND ((TI “law enforcement*” OR SU “law enforcement*” OR AB “law enforcement*”) OR (TI police OR SU police OR AB police))			
Web of Science	2026-04-10	(“Aerobic fitness” OR “cardiorespiratory fitness” OR “exercise test*” OR “fitness assessment*” OR “fitness test*” OR “functional performanc*” OR “functional performance test*” OR “grip strength” OR “physical abilit*” OR “physical capacit*” OR “physical endurance” OR “physical fitness” OR “physical fitness test*” OR “physical functional performanc*” OR “physical performanc*” OR “physical performance test*” OR “physical test*” OR “reference value*” OR “work sample test batter*”) AND (“Law enforcement*” OR police) (Topic)	English; Publication years 2025 or 2026	52	Updated search; Exported to EndNote
CINAHL (with Full Text)	2026-04-10	((TI “aerobic fitness” OR SU “aerobic fitness” OR AB “aerobic fitness”) OR (TI “cardiorespiratory fitness” OR SU “cardiorespiratory fitness” OR AB “cardiorespiratory fitness”) OR (TI “exercise test*” OR SU “exercise test*” OR AB “exercise test*”) OR (TI “fitness assessment*” OR SU “fitness assessment*” OR AB “fitness assessment*”) OR (TI “fitness test*” OR SU “fitness test*” OR AB “fitness test*”) OR (TI “functional performanc*” OR SU “functional performanc*” OR AB “functional performanc*”) OR (TI “functional performance test*” OR SU “functional performance test*” OR AB “functional performance test*”) OR (TI “grip strength” OR SU “grip strength” OR AB “grip strength”) OR (TI “physical abilit*” OR SU “physical abilit*” OR AB “physical abilit*”) OR (TI “physical capacit*” OR SU “physical capacit*” OR AB “physical capacit*”) OR (TI “physical endurance” OR SU “physical	English; Peer Reviewed; Date range (08/2025-04/2026)	9	Updated search; Exported to EndNote

		endurance” OR AB “physical endurance”) OR (TI “physical fitness” OR SU “physical fitness” OR AB “physical fitness”) OR (TI “physical fitness test*” OR SU “physical fitness test*” OR AB “physical fitness test*”) OR (TI “physical functional performanc*” OR SU “physical functional performanc*” OR AB “physical functional performanc*”) OR (TI “physical performanc*” OR SU “physical performanc*” OR AB “physical performanc*”) OR (TI “physical performance test*” OR SU “physical performance test*” OR AB “physical performance test*”) OR (TI “physical test*” OR SU “physical test*” OR AB “physical test*”) OR (TI “reference value*” OR SU “reference value*” OR AB “reference value*”) OR (TI “work sample test batter*” OR SU “work sample test batter*” OR AB “work sample test batter*”) OR (MH "Agility") OR (MH "Aerobic Capacity") OR (MH "Evaluation") OR (MH "Exercise Test+") OR (MH "Muscle Strength+") OR (MH "Physical Endurance+") OR (MH "Physical Fitness+") OR (MH "Physical Performance") OR (MH "Reference Values")) AND ((TI “law enforcement*” OR SU “law enforcement*” OR AB “law enforcement*”) OR (TI police OR SU police OR AB police) OR (MH "Police"))			
Citation searching and hand searching				1	
				Total	117
