# Supplementary material for: Methods Used to Assess Physical Abilities in Police Students: A Scoping Review
Source: Sports (Basel). 2026 Aug 18;14(8):356. doi: 10.3390/sports14080356 (PMC13517574; doi:10.3390/sports14080356)
Supplement: Supplementary file 1 [file sports-14-00356-s001.zip › Supplementary File S2.pdf]

Supplementary File S2. Reporting-scoring framework used to assess the completeness of test protocol reporting.
This supplementary file presents the complete reporting-scoring framework developed for the present review to evaluate the completeness of reporting of physical fitness test protocols. The framework consists of 20 items grouped into four domains: general information (3 points), equipment and setup (6 points), test protocol description (8 points), and reporting of test units and conventions (3 points), giving a maximum score of 20 points. Each applicable criterion was awarded 1 point when fully reported, 0.5 points when partially reported, and 0 points when not reported. Items that were not applicable to a specific test (N/A) or were explicitly referred to a previous publication or testing manual (R) were awarded 1 point. Total scores were classified as High (≥16), Medium (11–<16), or Low (<11) reporting standards.

Reporting criteria	Brief description	Points	Points
**1. General Information**		potential	achieved
**Title**	Title of the test		
**Objective of the Test**	Clearly stated what the test measured and its scientific purpose.		
**Test Rationale**	Explained *why* this particular protocol was chosen (e.g., relevance to previous studies).		
**2. Equipment and Setup**			
**Equipment and/or Software Details**	Specified manufacturer, model, calibration procedure, data acquisition, and analysis software (version).		
**Environmental Conditions**	Temperature, humidity, lighting, or other controlled parameters are reported.		
**Setup Description**	A clear diagram or photograph of the complex is provided.		
**Measurement Frequency and Resolution**	Sampling rate, precision, and data capture method were reported.		
**Data Processing Steps**	Filtering, normalization, or transformation methods were reported.		
**Calibration**	Timing, frequency, and method of calibration were reported.		
**3. Test Protocol**			
**Preparation and Warm-Up**	Pre-test routines, conditioning, or warm-up were described.		
**Procedure Step-by-Step**	Each stage of the test was described precisely in chronological order.		
**Positioning and Alignment**	Subject placement and reference points were described.		
**Instructions Given**	What participants were told to do.		
**Handling of Incorrect Attempts**	Specified what happens if a mistake (incorrect attempt) happens.		
**Number, Timing and Duration**	The duration of each trial and the number of trials were reported.		
**Rest Intervals**	Intra- and inter-test rest intervals were reported.		
**Safety Measures**	The protective or safety protocols were described.		
**4. Reporting**			
**Units and Conventions**	SI units and consistent terminology were used.		
**Reported Outcome Measure(s)**	Average result or best results		
**Deviations from Standard Protocols**	Potential test modifications were explained and justified.		
**TOTAL SCORE**			
