# Supplementary material for: Methods Used to Assess Physical Abilities in Police Students: A Scoping Review
Source: Sports (Basel). 2026 Aug 18;14(8):356. doi: 10.3390/sports14080356 (PMC13517574; doi:10.3390/sports14080356)
Supplement: Supplementary file 1 [file sports-14-00356-s001.zip › Supplementary File S3.pdf]

Supplementary File S3. Reporting standards assessment of the included studies.

This file provides the complete reporting standards assessment for each included study. Studies were evaluated using the reporting-scoring framework described in the Methods section, which assesses the completeness of reporting across four domains: test general information, equipment and setup, test protocol description, and test units and conventions. Domain-specific scores, total scores (maximum 20 points), and the corresponding reporting categories (High, Medium, or Low) are reported for all included studies.

**Authors**	**Reporting standards domains**				**Reporting standard**
	**Test general information** (3 points)	**Equipment and setup** (6 points)	**Test protocol description** (8 points)	**Test units and conventions** (3 points)	High ≥ 16<br>Medium 11–<16<br>Low < 11
Campbell et al. 2022 [1]	3/3	4/6	6/8	3/3	16/20
Gonzales et al. 2024 [2]	3/3	5/6	6/8	3/3	17/20
Marks et al. 2022 [3]	2/3	3/6	7/8	3/3	15/20
Jeknić et al. 2018 [4]	3/3	4/6	0/8	0/3	7/20
Annell et al. 2015 [5]	2/3	3/6	0/8	1/3	6/20
Jankovsky et al. 2013 [6]	1/3	3/6	0/8	1/3	5/20
Ramos & Massuça, 2025 [7]	3/3	5.5/6	8/8	3/3	19.5
Stojković et al. 2021 [8]	2/3	5/6	7.5/8	3/3	17.5
Thielgen & Schade 2024 [9]	3/3	4/6	6/8	3/3	16
Bloodgood et al. 2021 [10]	3/3	6/6	8/8	3/3	20
Bloodgood et al. 2020 [11]	3/3	5/6	7/8	3/3	18
Campbell et al. 2024 [12]	3/3	6/6	8/8	3/3	20
Campbell et al. 2024 [13]	3/3	6/6	8/8	3/3	20
Cervantes-Sanabria et al. 2025 [14]	2/3	5/6	6/8	3/3	16
Cesario et al. 2018 [15]	3/3	5/6	7/8	3/3	18
Cocke et al. 2016 [16]	3/3	6/6	8/8	3/3	20
Collins et al. 2022 [17]	3/3	6/6	7/8	3/3	19
Crawley et al. 2016 [18]	3/3	3/6	8/8	3/3	17
Čvorović et al. 2021 [19]	3/3	5/6	8/8	3/3	19
Dawes et al. 2019 [20]	3/3	5/6	6/8	3/3	17
Dawes et al. 2022 [21]	3/3	5/6	8/8	3/3	19
Dimitrijević et al. 2014 [22]	3/3	4/6	6.5/8	3/3	16.5
Dos Santos et al. 2023 [23]	3/3	6/6	8/8	3/3	20
Dulla et al. 2024 [24]	3/3	5/6	7/8	3/3	18
Freitas & Massuça 2025 [25]	3/3	6/6	8/8	3/3	20
Freitas, Ramos & Massuça 2025 [26]	3/3	6/6	8/8	3/3	20

Heredia et al. 2024 [27]	3/3	5/6	7/8	3/3	18
Hernandez et al. 2021 [28]	3/3	6/6	7/8	3/3	19
Hernandez et al. 2021 [29]	3/3	6/6	8/8	3/3	20
Iorga et al. 2024 [30]	3/3	5/6	2/8	2/3	12
Janković 2023 [31]	3/3	6/6	7/8	3/3	19
Janković 2015 et al. [32]	3/3	5/6	7/8	3/3	18
Janković & Koropanovski 2024 [33]	3/3	6/6	7/8	3/3	19
Koropanovski et al. 2020 [34]	2.5/3	2.5/6	5/8	2/3	12
Koropanovski et al. 2022 [35]	2.5/3	5/6	4/8	3/3	14.5
Korre et al. 2019 [36]	2/3	5/6	3/8	3/3	13
Kukić et al. 2023 [37]	3/3	6/6	8/8	3/3	20
Kukić, Janković & Koropanovski 2023 [38]	3/3	5/6	8/8	3/3	19
Kukić et al. 2019 [39]	3/3	5/6	8/8	2/3	18
Kukić et al. 2019 [40]	3/3	5/6	7/8	3/3	18
Kukić et al. 2020 [41]	3/3	5/6	6/8	3/3	17
Kukić et al. 2022 [42]	3/3	6/6	8/8	3/3	20
Kukić et al. 2024 [43]	3/3	5/6	7/8	3/3	18
Lagestad & Van den Tillaar 2014 [44]	3/3	5/6	5/8	2/3	15
Lockie et al. 2023 [45]	3/3	6/6	6/8	3/3	18
Lockie 2024 [46]	3/3	6/6	7/8	3/3	19
Lockie 2025 [47]	3/3	6/6	7/8	3/3	19
Lockie et al. 2019 [48]	3/3	6/6	7/8	3/3	19
Lockie et al. 2022 [49]	3/3	5/6	8/8	3/3	19
Lockie et al. 2021 [50]	3/3	6/6	7/8	3/3	19
Lockie et al. 2018 [51]	3/3	6/6	6/8	3/3	18
Lockie et al. 2020 [52]	3/3	6/6	8/8	3/3	20
Lockie et al. 2020 [53]	3/3	5/6	6.5/8	3/3	17.5
Lockie et al. 2021 [54]	3/3	5/6	8/8	3/3	19
Lockie et al. 2020 [55]	3/3	6/6	8/8	3/3	20
Lockie et al. 2020 [56]	3/3	5/6	7/8	3/3	18
Lockie et al. 2021 [57]	3/3	5/6	6/8	3/3	17
Lockie et al. 2022 [58]	3/3	6/6	7/8	3/3	19
Lockie et al. 2018 [59]	3/3	5/6	5/8	3/3	16
Lockie et al. 2020 [60]	3/3	4/6	7.5/8	3/3	17.5
Lockie et al. 2020 [61]	3/3	5/6	7/8	3/3	18
Lockie et al. 2021 [62]	3/3	6/6	8/8	3/3	20

Lockie et al. 2021 [63]	3/3	6/6	7/8	3/3	19
Lockie et al. 2024 [64]	3/3	6/6	8/8	3/3	20
Lockie et al. 2024 [65]	3/3	5/6	8/8	3/3	19
Lockie et al. 2020 [66]	3/3	6/6	7/8	3/3	19
Lockie et al. 2025 [67]	3/3	5/6	6/8	3/3	17
Martinez & Abel 2021[68]	2/3	6/6	7/8	1/3	16
Martinez et al. 2022 [69]	3/3	5/6	4/8	1,5/3	13.5
Massuça et al. 2023 [70]	2.5/3	5/6	5/8	3/3	15.5
Maupin et al. 2024 [71]	2.5/3	5/6	6/8	3/3	16,5
Maupin et al. 2020 [72]	3/3	5/6	6/8	3/3	17
Merrigan et al. 2022 [73]	3/3	5/6	8/8	3/3	19
Moreno et al. 2019 [74]	3/3	6/6	6/8	3/3	18
Murphy et al. 2024 [75]	3/3	4/6	3/8	3/3	13
Orr et al. 2016 [76]	3/3	5/6	6/8	3/3	17
Orr et al. 2017 [77]	3/3	5/6	2.5/8	3/3	13.5
Orr et al. 2021 [78]	2.5/3	5/6	3.5/8	3/3	14
Orr et al. 2018 [79]	2.5/3	5/6	8/8	2.5/3	18
Orr et al. 2020 [80]	3/3	5/6	7/8	3/3	18
Orr, Ford & Stierli 2016 [81]	3/3	5/6	6/8	3/3	17
Papadakis et al. 2021 [82]	3/3	5/6	5/8	3/3	16
Poirier et al. 2021 [83]	3/3	6/6	5/8	3/3	17
Poirier et al. 2022 [84]	3/3	4/6	6/8	3/3	16
Poirier et al. 2023 [85]	3/3	5/6	4.5/8	3/3	15.5
Schram et al. 2024 [86]	3/3	5/6	5.5/8	3/3	16.5
Shusko et al. 2017 [87]	1.5/3	5/6	2/8	2/3	10.5
Sousa-Sá et al. 2023 [88]	2/3	5/6	5/8	3/3	15
Sutton et al. 2025 [89]	3/3	5/6	5/8	3/3	16
Tomes et al. 2020 [90]	1.5/3	4/6	5/8	3/3	13.5
Wu 2015 [91]	2/3	5/6	2/8	2/3	11
Koropanovski et al. 2022 [92]	3/3	6/6	7/8	3/3	19
Lockie et al. 2022 [93]	3/3	6/6	6/8	3/3	18
Koedijk et al. 2023 [94]	3/3	6/6	6/8	3/3	18
Kolarević et al. 2014 [95]	3/3	5/6	3.5/8	1.5/3	13
Henze et al. 2024 [96]	3/3	5/6	7/8	2/3	17
Krugly et al. 2023 [97]	3/3	5/6	8/8	3/3	19
Canetti et al. 2025 [98]	3/3	6/6	8/8	3/3	20

Jia 2025 [99]	3/3	6/6	5/8	3/3	17
Janković & Dopsaj 2022 [100]	3/3	5/6	7/8	3/3	18
Němá et al. 2025 [101]	3/3	5/6	4/8	2/3	14

References

1. Campbell, P.; Maupin, D.; Lockie, R.G.; Dawes, J.J.; Simas, V.; Canetti, E.; Schram, B.; Orr, R. The Development of Normative Fitness Data and Analyzing the Relationships Between 20MSFT and 2.4-km Run Performance in Australian Police Recruits. *J. Strength Cond. Res.* **2022**, *38*, 1951-1958.
2. Gonzales, S.M.; Orr, R.M.; Coburn, J.W.; Hoffmann, M.D.; Kennedy, K.; Dawes, J.J.; Lockie, R.G. A Retrospective Analysis of Southeastern US Police Recruit Health and Fitness From 4 Points in Time Within a 16-Year Period: Implications for Physical Fitness Training. *J. Strength Cond. Res.* **2024**, *38*, 1118-1126.
3. Marks, D.; Merrigan, J.J.; Martin, J. Do baseline physical fitness measures predict law enforcement academy graduation? *Work.* **2022**, *72*, 263-269.
4. Jeknić, V.; Stojković, M.; Bacetić, N. Fitness level comparison between police college freshman and senior students. *Int. J. Phys. Educ. Sports Health.* **2018**, *5*, 99-104.
5. Annell, S.; Lindfors, P.; Sverke, M. Police selection–implications during training and early career. *Policing: Int. J. Police Strat. Manag.* **2015**, *38*, 221-238.
6. Jankovsky, P.; Sedlacek, J.; Balint, G. The level comparision of somatic parameters and general physical fitness of Bratislava Police Academy male students. *Gymnasium.* **2013**, *14*.
7. Ramos, N.; Massuça, L.M. Reliability of Police Physical Tasks and Fitness Predictors. *Appl. Sci.* **2025**, *15*, 9271.
8. Stojković, M.; Kukić, F.; Nedeljković, A.; Orr, R.M.; Dawes, J.J.; Čvorović, A.; Jeknić, V. Effects of a physical training programme on anthropometric and fitness measures in obese and overweight police trainees and officers. *S. Afr. J. Res. Sport Phys. Educ. Recreation*. **2021**, *43*, 63-76.
9. Thielgen, M.M.; Schade, S. Physical Job Demands in Police Officer Selection and Training: Normative Data and Predictive Validity—A Research Note. *J. Police Crim. Psychol.* **2024**, *39*, 660-670.
10. Bloodgood, A.M.; Dawes, J.J.; Orr, R.M.; Stierli, M.; Cesario, K.A.; Moreno, M.R.; Dulla, J.M.; Lockie, R.G. Effects of sex and age on physical testing performance for law enforcement agency candidates: Implications for academy training. *J. Strength Cond. Res.* **2021**, *35*, 2629-2635.
11. Bloodgood, A.M.; Moreno, M.R.; Cesario, K.A.; McGuire, M.B.; Lockie, R.G. An investigation of seasonal variations in the fitness test performance of law enforcement recruits. *Facta Universitatis, Series: Physical Education and Sport.* **2020**, 271-282.
12. Campbell, P.; Maupin, D.; Lockie, R.G.; Dawes, J.J.; Simas, V.; Canetti, E.; Schram, B.; Orr, R. Determining the Changes in Law Enforcement Recruit Aerobic Fitness Using the 2.4-km Run and 20-m Multistage Fitness Test. Does the Type of Test Matter? *J. Strength Cond. Res.* **2024**, *38*, 1111-1117.
13. Campbell, P.; Maupin, D.; Lockie, R.G.; Dawes, J.J.; Simas, V.; Canetti, E.; Schram, B.; Orr, R. Evaluating the variability between 20-m multistage fitness test estimating equations in law enforcement recruits. *J. Strength Cond. Res.* **2024**, *38*, 742-748.
14. Cervantes-Sanabria, J.; Camacho-Sánchez, G.; Hernández-Elizondo, J.; Carazo-Vargas, P.; Brazo-Sayavera, F.J.; Castillo-Hernández, I.M.; Salicetti-Fonseca, A. Age, sex and adiposity correlates of physical fitness in Costa Rican police academy cadets. *Pensar en Movimiento: Revista de ciencias del ejercicio y la salud.* **2025**, *23*, 72-94.
15. Cesario, K.; Dulla, J.; Blood Good, A.; Moreno, M.R.; Dawes, J.J.; Lockie, R.G. Relationships between assessments in a physical ability test for law enforcement: Is there redundancy in certain assessments? *Int. J. Exerc. Sci.* **2018**, *11*, 1063-1073.

16. Cocke, C.; Dawes, J.; Orr, R.M. The use of 2 conditioning programs and the fitness characteristics of police academy cadets. *J. Athl. Train.* **2016**, *51*, 887-896.
17. Collins, K.S.; Christensen, B.K.; Orr, R.M.; Dulla, J.M.; Dawes, J.J.; Lockie, R.G. Analysis of total and segmental body composition relative to fitness performance measures in law enforcement recruits. *Int. J. Exerc. Sci.* **2022**, *15*, 245.
18. Crawley, A.A.; Sherman, R.A.; Crawley, W.R.; Cosio-Lima, L.M. Physical fitness of police academy cadets: Baseline characteristics and changes during a 16-week academy. *J. Strength Cond. Res.* **2016**, *30*, 1416-1424.
19. Čvorović, A.; Kukić, F.; Orr, R.M.; Dawes, J.J.; Jeknić, V.; Stojković, M. Impact of a 12-week postgraduate training course on the body composition and physical abilities of police trainees. *J. Strength Cond. Res.* **2021**, *35*, 826-832.
20. Dawes, J.; Lockie, R.G.; Orr, R.M.; Kornhauser, C.; Holmes, R. Initial fitness testing scores as a predictor of police academy graduation. *J. Aust. Strength Cond.* **2019**, *27*, 30-37.
21. Dawes, J.J.; Scott, J.; Canetti, E.F.; Lockie, R.G.; Schram, B.; Orr, R.M. Profiling the New Zealand police trainee physical competency test. *Front. Public Health.* **2022**, *10*, 821451.
22. Dimitrijević, R.; Koropanovski, N.; Dopsaj, M.; Vučković, G.; Janković, R. The influence of different physical education programs on police students' physical abilities. *Policing: Int. J. Police Strateg. Manag.* **2014**, *37*, 794-808.
23. Dos Santos, M.L.; Thompson, M.; Dinyer-McNeely, T.; Torrence, T.; Lockie, R.G.; Orr, R.M.; Dawes, J.J. Differences and relationships between push-up and sit-up variations among male law enforcement cadets. *J. Strength Cond. Res.* **2023**, *37*, 1865-1869.
24. Dulla, J.; Lockie, R.; Orr, R.; Schram, B.; Canetti, E. A profile of two different applicant test batteries with regards to sex and age in successful law enforcement applicants. *Work.* **2024**, *77*, 1359-1368.
25. Freitas, J.D.; Massuça, L.M. Fitness Profile of Police Officers from Rapid Intervention Teams of the Lisbon Metropolitan Command. *J. Funct. Morphol. Kinesiol.* **2025**, *10*, 90.
26. Freitas, J.D.; Ramos, S.A.; Massuça, L.M. Reliability of Critical Fitness Tests for Police Officers. *Appl. Sci.* **2025**, *15*, 405.
27. Heredia, C.E.; Dawes, J.J.; Dulla, J.M.; Orr, R.M.; Lockie, R.G. Shoulder Taps: Relationships Between a New Movement Screening Assessment with Body Composition and Physical Fitness in Law Enforcement Recruits. *Int. J. Exerc. Sci.* **2024**, *17*, 702.
28. Hernandez, E.; Dawes, J.J.; Dulla, J.; Orr, R.; Lockie, R.G. Fitness percentile rank data for female law enforcement recruits in the USA. *Facta Universitatis, Series: Physical Education and Sport.* **2021**, 109-118.
29. Hernandez, E.; Dawes, J.J.; Orr, R.M.; Dulla, J.M.; Lockie, R.G. Are there differences in fitness between recruits from larger (hosting) and smaller (participating) law enforcement agencies? *Int. J. Exerc. Sci.* **2021**, *14*, 885.
30. Iorga, V.; De Hillerin, P.-J.; Dancescu, D.; Geanta, V.A. Importance of integrating psychophysical training into the holistic training program for law enforcement *J. Phys. Educ. Sport.* **2024**, *24*, 1434-1444.
31. Janković, R. Correlation of basic and specific physical abilities: a study on the obstacle course for assessing specific agility of police officers. *SportLogia.* **2023**, *19*.

32. Janković, R.Z.; Dopsaj, M.; Dimitrijević, R.; Savković, M.; Koropanovski, N.; Vučković, G. Validity and reliability of the test for assessment of specific physical abilities of police officers in the anaerobic-lactate work regime. *Facta Universitatis, Series: Physical Education and Sport.* **2015**, 19-32.
33. Janković, R.; Koropanovski, N. Correlation of Morphological Characteristics with Police Specific Fitness Test in Police Students. *SportLogia.* **2024**, *20*.
34. Koropanovski, N.; Kukić, F.; Janković, R.; Dimitrijević, R.; Dawes, J.J.; Lockie, R.G.; Dopsaj, M. Impact of physical fitness on recruitment and its association to study outcomes of police students. *S. Afr. J. Res. Sport Phys. Educ. Recreation.* **2020**, *42*, 23-34.
35. Koropanovski, N.; Kukić, F.; Janković, R.; Kolarević, D.; Subošić, D.; Orr, R.M. Intellectual potential, personality traits, and physical fitness at recruitment: Relationship with academic success in police studies. *Sage Open.* **2022**, *12*, 21582440221079932.
36. Korre, M.; Loh, K.; Eshleman, E.J.; Lessa, F.S.; Porto, L.; Christophi, C.A.; Kales, S.N. Recruit fitness and police academy performance: a prospective validation study. *Occup. Med.* **2019**, *69*, 541-548.
37. Kukić, F.; Janković, R.; Dawes, J.J.; Orr, R.; Koropanovski, N. Effects of occupational load on the acceleration, change of direction speed, and anaerobic power of police officers. *J. Strength Cond. Res.* **2023**, *37*, 1237-1243.
38. Kukić, F.; Janković, R.; Koropanovski, N. Association of physical abilities to academic success in police students. *SportLogia.* **2023**, *19*.
39. Kukic, F.; Jeknic, V.; Dawes, J.J.; Orr, R.M.; Stojkovic, M.; Cvorovic, A. Effects of training and a semester break on physical fitness of police trainees. *Kinesiology* **2019**, *51*, 161-169.
40. Kukić, F.; Koropanovski, N.; Janković, R.; Dopsaj, M. Effects of specialized physical education and additional aerobic training on aerobic endurance of police students. *Hum. Sport. Med.* **2019**, *19*, 58-64.
41. Kukić, F.; Lockie, R.G.; Vesković, A.; Petrović, N.; Subošić, D.; Spasić, D.; Paspalj, D.; Vulin, L.; Koropanovski, N. Perceived and measured physical fitness of police students. *Int. J. Environ. Res. Public Health.* **2020**, *17*, 7628.
42. Kukić, F.; Orr, R.; Marković, M.; Dawes, J.J.; Čvorović, A.; Koropanovski, N. Factorial and Construct Validity of Sit-Up Test of Different Durations to Assess Muscular Endurance of Police Students. *Sustainability.* **2022**, *14*, 13630.
43. Kukić, F.; Zlojutro, N.; Paspalj, D.; Bajić, S.; Kovačević, S.; Vulin, L.; Rađević, N.; Koropanovski, N. Effects of specific RAMP protocol phase on change of direction speed of police students. *J. Funct. Morphol. Kinesiol.* **2024**, *9*, 194.
44. Lagestad, P.; Van den Tillaar, R. A comparison of training and physical performance of police students at the start and the end of three-year police education. *J. Strength Cond. Res.* **2014**, *28*, 1394-1400.
45. Lockie, R.; Dawes, J.J.; Sakura, T.; Schram, B.; Orr, R.M. Relationships between physical fitness assessment measures and a workplace task-specific physical assessment among police officers: A retrospective cohort study. *J. Strength Cond. Res.* **2023**, *37*, 678-683.
46. Lockie, R.G. 20-m Multistage Fitness Test Normative Percentile Rank Data for Law Enforcement Recruits. Sportlogia, **2024**, 21(1), 12-23. https://doi.org/10.63356/spl.2025.002
47. Lockie, R.G. Percentile Rank Data for the Countermovement Vertical Jump Measured by a Jump-and-Reach Device in Law Enforcement Recruits. *SportLogia.* **2025**, *21*.

48. Lockie, R.G.; Balfany, K.; Bloodgood, A.M.; Moreno, M.R.; Cesario, K.A.; Dulla, J.M.; Dawes, J.J.; Orr, R.M. The influence of physical fitness on reasons for academy separation in law enforcement recruits. *Int. J. Environ. Res. Public Health.* **2019**, *16*, 372.
49. Lockie, R.G.; Beitzel, M.M.; Dulla, J.M.; Dawes, J.J.; Orr, R.M.; Hernandez, J.A. Between-sex differences in the Work Sample Test Battery performed by law enforcement recruits: Implications for training and potential job performance. *J. Strength Cond. Res.* **2022**, *36*, 1310-1317.
50. Lockie, R.G.; Carlock, B.N.; Ruvalcaba, T.J.; Dulla, J.M.; Orr, R.M.; Dawes, J.J.; McGuire, M.B. Skeletal muscle mass and fat mass relationships with physical fitness test performance in law enforcement recruits before academy. *J. Strength Cond. Res.* **2021**, *35*, 1287-1295.
51. Lockie, R.G.; Dawes, J.J.; Balfany, K.; Gonzales, C.E.; Beitzel, M.M.; Dulla, J.M.; Orr, R.M. Physical fitness characteristics that relate to Work Sample Test Battery performance in law enforcement recruits. *Int. J. Environ. Res. Public Health* **2018**, *15*, 2477.
52. Lockie, R.G.; Dawes, J.J.; Dulla, J.M.; Orr, R.M.; Hernandez, E. Physical fitness, sex considerations, and academy graduation for law enforcement recruits. *J. Strength Cond. Res.* **2020**, *34*, 3356-3363.
53. Lockie, R.G.; Dawes, J.J.; D MACLEAN, N.; Pope, R.P.; Holmes, R.J.; Kornhauser, C.L.; Orr, R.M. The impact of formal strength and conditioning on the fitness of law enforcement recruits: A retrospective cohort study. *Int. J. Exerc. Sci.* **2020**, *13*, 1615.
54. Lockie, R.G.; Dawes, J.J.; Moreno, M.R.; Cesario, K.A.; Balfany, K.; Stierli, M.; Dulla, J.M.; Orr, R.M. Relationship between the 20-m multistage fitness test and 2.4-km run in law enforcement recruits. *J. Strength Cond. Res.* **2021**, *35*, 2756-2761.
55. Lockie, R.G.; Dawes, J.J.; Moreno, M.R.; McGuire, M.B.; Ruvalcaba, T.J.; Bloodgood, A.M.; Dulla, J.M.; Orr, R.M. We need you: Influence of hiring demand and modified applicant testing on the physical fitness of law enforcement recruits. *Int. J. Environ. Res. Public Health.* **2020**, *17*, 7512.
56. Lockie, R.G.; Dawes, J.J.; Orr, R.M.; Dulla, J.M. Recruit fitness standards from a large law enforcement agency: Between-class comparisons, percentile rankings, and implications for physical training. *J. Strength Cond. Res.* **2020**, *34*, 934-941.
57. Lockie, R.G.; Dawes, J.J.; Orr, R.M.; Dulla, J.M. Physical fitness: Differences between initial hiring to academy in law enforcement recruits who graduate or separate from academy. *Work.* **2021**, *68*, 1081-1090.
58. Lockie, R.G.; Dawes, J.J.; Orr, R.M.; Dulla, J.M. The bigger they are: Relationships between body height and mass with the body drag task in law enforcement recruits. *Int. J. Exerc. Sci.* **2022**, *15*, 570.
59. Lockie, R.G.; Dawes, J.J.; Orr, R.M.; Stierli, M.; Dulla, J.M.; Orjalo, A.J. Analysis of the effects of sex and age on upper-and lower-body power for law enforcement agency recruits before academy training. *J. Strength Cond. Res.* **2018**, *32*, 1968-1974.
60. Lockie, R.G.; Hernandez, J.A.; Moreno, M.R.; Dulla, J.M.; Dawes, J.J.; Orr, R.M. 2.4-km run and 20-m multistage fitness test relationships in law enforcement recruits after academy training. *J. Strength Cond. Res.* **2020**, *34*, 942-945.
61. Lockie, R.G.; Moreno, M.R.; McGuire, M.B.; Ruvalcaba, T.J.; Bloodgood, A.M.; Dulla, J.M.; Orr, R.M.; Dawes, J.J. Relationships between isometric strength and the 74.84-kg (165-lb) body drag test in law enforcement recruits. *J. Hum. Kinet.* **2020**, *74*, 5.
62. Lockie, R.G.; Moreno, M.R.; Rodas, K.A.; Dulla, J.M.; Orr, R.M.; Dawes, J.J. With great power comes great ability: Extending research on fitness characteristics that influence Work Sample Test Battery performance in law enforcement recruits. *Work.* **2021**, *68*, 1069-1080.
63. Lockie, R.G.; Orr, R.M.; Dulla, J.; Dawes, J.J. Relationships between Absolute and Relative Lower-Body Power and Foot Pursuit Ability in Law Enforcement Recruits. *Int. J. Exerc. Sci.* **2021**, *14*, 1078-1089.

64. Lockie, R.G.; Orr, R.M.; Kennedy, K.; Dawes, J.J. Introduction of an applicant Job-Related Task Assessment (JTA) and the effects on the health and fitness of police recruits. *Work.* **2024**, *77*, 1401-1413.
65. Lockie, R.G.; Orr, R.M.; Sanchez, K.J.; Gonzales, S.M.; Viramontes, E.; Kennedy, K.; Dawes, J.J. Relationships between physical ability test performance and fitness in recruits from a Southeastern US police department. *J. Strength Cond. Res.* **2024**, *38*, 1472-1478.
66. Lockie, R.G.; Ruvalcaba, T.R.; Stierli, M.; Dulla, J.M.; Dawes, J.J.; Orr, R.M. Waist circumference and waist-to-hip ratio in law enforcement agency recruits: Relationship to performance in physical fitness tests. *J. Strength Cond. Res.* **2020**, *34*, 1666-1675.
67. Lockie, R.G.; Young, M.A.; Lanham, S.N.; Orr, R.M.; Dawes, J.J.; Nagel, T.R. Retrospective Analysis of Grip and Index Finger Strength, Job-Specific Scenario, and Shooting Performance in Incumbent Law Enforcement Personnel and Cadets. *J. Strength Cond. Res.* **2025**, *39*, e581-e588.
68. Martinez, G.J.; Abel, M.G. Effect of a law enforcement academy training program on validated fitness outcomes of cadets. *J. Strength Cond. Res.* **2021**, *35*, 955-962.
69. Martinez, G.J.; Ma, X.; Best, S.; Johnson, B.F.; Abel, M.G. Implementation of High Intensity Interval Training and Autoregulatory Progressive Resistance Exercise in a Law Enforcement Training Academy. *Int. J. Exerc. Sci.* **2022**, *15*, 1246.
70. Massuça, L.M.; Monteiro, L.; Coutinho, G.; Santos, V. Four-year training course for police officers (CFOP) and fitness outcomes of police academy cadets: A cohort study from 2004 to 2020. In Proceedings of the Healthcare. **2023**; p. 2901.
71. Maupin, D.; Canetti, E.F.; Schram, B.; Dulla, J.M.; Lockie, R.G.; Dawes, J.J.; Orr, R.M. Law enforcement recruit fitness: Changes across the fitness spectrum. *Work.* **2024**, *79*, 947-963.
72. Maupin, D.; Schram, B.; Canetti, E.; Dawes, J.; Lockie, R.; Orr, R. Developing Fitness of Law Enforcement Recruits during Academy Training. *Sustainability*. **2022,** 12, 7944.
73. Merrigan, J.J.; Burke, A.A.; Eddo, O.O.; Kearney, J.; Marks, D.; Martin, J.R. Upper body push to pull ratios in law enforcement officer recruits. *Work.* **2022**, *73*, 1167-1174.
74. Moreno, M.R.; Dulla, J.; Dawes, J.; Orr, R.M.; Cesario, K.A.; Lockie, R.G. Lower-body power and its relationship with body drag velocity in law enforcement recruits. *Int. J. Exerc. Sci.* **2019**, *12*, 847-858.
75. Murphy, M.C.; Merrick, N.; Mosler, A.B.; Allen, G.; Chivers, P.; Hart, N.H. Cardiorespiratory fitness is a risk factor for lower-limb and back injury in law enforcement officers commencing their basic training: a prospective cohort study. *Res. Sports Med.* **2024**, *32*, 511-523.
76. Orr, R.; Pope, R.; Peterson, S.; Hinton, B.; Stierli, M. Leg power as an indicator of risk of injury or illness in police recruits. *Int. J. Environ. Res. Public Health.* **2016**, *13*, 237.
77. Orr, R.; Pope, R.; Stierli, M.; Hinton, B. Grip strength and its relationship to police recruit task performance and injury risk: A retrospective cohort study. *Int. J. Environ. Res. Public Health.* **2017**, *14*, 941.
78. Orr, R.M.; Canetti, E.; Movshovich, J.; Lockie, R.; Dawes, J.; Schram, B. Profiling the New Zealand police physical appraisal test. *Int. J. Emerg. Serv.* **2021**, *10*, 266-275.
79. Orr, R.M.; Dawes, J.J.; Pope, R.; Terry, J. Assessing differences in anthropometric and fitness characteristics between police academy cadets and incumbent officers. *J. Strength Cond. Res.* **2018**, *32*, 2632-2641.

80. Orr, R.M.; Ferguson, D.; Schram, B.; Dawes, J.J.; Lockie, R.; Pope, R. The relationship between aerobic test performance and injuries in police recruits. *Int. J. Exerc. Sci.* **2020**, *13*, 1052.
81. Orr, R.M.; Ford, K.; Stierli, M. Implementation of an ability-based training program in police force recruits. *J. Strength Cond. Res.* **2016**, *30*, 2781-2787.
82. Papadakis, Z.; Stamatis, A.; Kukic, F.; Koropanovski, N. Moving past the one-size-fits-all education-training model of police academies to the self-prescribed individualized exercise prescription model. *Int. J. Environ. Res. Public Health.* **2021**, *18*, 11676.
83. Poirier, S.; Gendron, A.; Gendron, P.; Lajoie, C. Fitness components associated with performance of a law enforcement physical employment standard in police cadets. *J. Sports Med. Phys. Fitness.* **2021**, *62*, 981-989.
84. Poirier, S.; Gendron, A.; Trudeau, F.; Lajoie, C. Cardiorespiratory fitness in police recruits: Assessing the validity of the 20-meter shuttle run test for recruitment purposes. *Work.* **2022**, *71*, 1193-1201.
85. Poirier, S.; Houle, J.; Lajoie, C.; Trudeau, F. Cardiorespiratory fitness of police recruits: Normative reference values and temporal trend. *J. Strength Cond. Res.* **2023**, *37*, 207-212.
86. Schram, B.; Kukić, F.; Janković, R.; Dimitrijević, R.; Žigić, G.; Orr, R.; Koropanovski, N. Effects of a single-day pre-academy physical test training session on physical fitness scores of police candidates. *Work.* **2024**, *77*, 1369-1375.
87. Shusko, M.; Benedetti, L.; Korre, M.; Eshleman, E.J.; Farioli, A.; Christophi, C.A.; Kales, S.N. Recruit fitness as a predictor of police academy graduation. *Occup. Med.* **2017**, *67*, 555-561.
88. Sousa-Sá, E.; Pereira, S.; Júdice, P.B.; Monteiro, L.; Massuça, L.M. COVID-19 Social Restrictions' Impact on the Health-Related Physical Fitness of the Police Cadets. In Proceedings of the *Healthcare*. **2023**; p. 1949.
89. Sutton, V.R.; Murphy, M.C.; McCaskie, C.J.; Chivers, P.T.; Hart, N.H.; Cochrane Wilkie, J.L.; Allen, G.; Dalla Via, J. The Association of Body Composition and Musculoskeletal Characteristics with Police Recruit Performance: A Cross-Sectional Study. *J. Funct. Morphol. Kinesiol.* **2025**, *10*, 132.
90. Tomes, C.; Schram, B.; Pope, R.; Orr, R. What is the impact of fitness on injury risk during police academy training? A retrospective cohort study. *BMC Sports Sci. Med. Rehabil.* **2020**, *12*, 39.
91. Wu, Y.-N.; Hallbourg, K.W.; Collins, S.M. Changes of general fitness and muscle properties following police cadet training. *J. Phys. Ther. Sci.* **2015**, *27*, 2783-2786.
92. Koropanovski, N.; Orr, R.M.; Dopsaj, M.; Heinrich, K.M.; Dawes, J.J.; Kukic, F. Effects of maximal and submaximal anaerobic and aerobic running on subsequent change-of-direction speed performance among police students. *Biology.* **2022**, *11*, 767.
93. Lockie, R.G.; Moreno, M.R.; Dawes, J.J.; Orr, R.M.; Rodas, K.A.; Dulla, J.M. An analysis of the body drag test in law enforcement recruits with consideration to current population demographics. *Int. J. Exerc. Sci.* **2022**, *15*, 276.
94. Koedijk, M.; Hutter, R.; Renden, P.G.; Kleygrewe, L.; Oudejans, R.R. Fitness testing at police academies: Optimal fitness for duty. *Int. J. Police Sci. Manag.* **2023**, *25*, 170-182.
95. Kolarević, D.; Dimitrijevic, R.; Vučković , G.; Koropanovski, N.; Dopsaj, M. Relations between psychological characteristics and physical abilities in a sample of female police candidates. The Open Sports Sciences Journal, 2014, 7, (Suppl-1, M5) 22-28.

96. Henze, K.M.; Klausen, L.K.; Martín, G.F.; Dillern, T. Physical Fitness and Use-of-Force Performance for Police Students: The Impact of Body Height, Body Mass, Age, and Gender. *Nord. J. Stud. Policing.* **2024**, *11*, 1-23.
97. Krugly, S.; Bjärsholm, D.; Jansson, A.; Rosendal Hansen, A.; Hansson, O.; Brehm, K.; Datmo, A.; Hafsteinsson Östenberg, A.; Vikman, J. A retrospective study of physical fitness and mental health among police students in Sweden. *Police J.* **2023**, *96*, 430-450.
98. Canetti, E.F.D.; Schram, B.; Pope, R.; Lockie, R.G.; Dawes, J.J.; Campbell, P.; Orr, R. Test-Retest Reliability of the 20-Metre Shuttle Run Test in a Cohort of Police Trainees. *SportLogia.* **2025**, *21*, 1-7, doi:10.63356/spl.2025.005.
99. Jia, D. Graduation-stage physical fitness as a predictor of operational performance and injury risk among police recruits: a prospective cohort study. *BMJ Open Sport Exerc. Med.* **2025**, *11*, e002871, doi:10.1136/bmjsem-2025-002871.
100. Jankovic, R.; Dopsaj, M. Sprint running, agility and anaerobic endurance: Standards for the students at the University of Criminal Investigation and Police Studies. *Nauka bezbednost policija.* **2022**, *27*, 45-58, doi:10.5937/nabepo27-36046.
101. Němá, K.; Kozák, T.; Ligday, M.; Rydzik, Ł. Sport Mont 23 (2025) 3: 13-18 13 Physical Fitness of Police Force Applicants: Trends from 2010 to 2023. *Sport Mont.* **2025**, *23*, 13-18, doi:10.26773/smj.251002.
