# Supplementary material for: Methods Used to Assess Physical Abilities in Police Students: A Scoping Review
Source: Sports (Basel). 2026 Aug 18;14(8):356. doi: 10.3390/sports14080356 (PMC13517574; doi:10.3390/sports14080356)
Supplement: Supplementary file 1 [file sports-14-00356-s001.zip › Supplementary File S4.pdf]

Supplementary File S4. Reporting of reliability and validity of the studies included.

This file provides a complete report of the reliability and validity assessments for each included study. Reliability was evaluated solely based on the completeness of reporting within each study. Specifically, reliability was scored as: 1 = not reported; 2 = not assessed but supported by reliability evidence from previous studies; and 3 = assessed and reported within the study. The assessment focused only on whether reliability information was reported and did not evaluate the quality of the reported reliability estimates. Consequently, reliability cannot be determined with certainty, as tests may have demonstrated reliability in previous research even if this was not assessed or reported in the included study. Validity was recorded only when the primary aim of the study was to validate the test(s). Validity was coded as 1 = tested and 0 = not tested. The assessment did not consider whether the test(s) had been validated in previous studies. Therefore, the absence of reported validation should not be interpreted as evidence that a test lacks validity, as the test(s) may have been validated previously but not within the scope of the included study.

Author	General fitness tests		Work-related police tests	
	Reliability	Validity	Reliability	Validity
Campbell et al. 2022 [1]	2	0		
Gonzales et al. 2024 [2]	1	0	1	0
Marks et al. 2022 [3]	1	0	1	0
Jeknić et al. 2018 [4]	1	0		
Annell et al. 2015 [5]	1	0		
Jankovsky et al. 2013 [6]	1	0		
Ramos & Massuça 2025 [7]	1	0	3	0
Stojkovic et al. 2021 [8]	1	0		
Thielgen & Schade 2024 [9]	1	0	1	0
Bloodgood et al. 2021 [10]	1	0		
Bloodgood et al. 2020 [11]	1	0		
Campbell et al. 2024 [12]	2	0		
Campbell et al. 2024 [13]	2	0		
Cervantes-Sanabria et al. 2025 [14]	1	0		
Cesario et al. 2018 [15]	1	0		
Cocke et al. 2016 [16]	1	0		
Collins et al. 2022 [17]	1	0		
Crawley et al. 2016 [18]	1	0		
Čvorović et al. 2021 [19]	1	0		
Dawes et al. 2019 [20]	1	0		
Dawes et al. 2022 [21]			1	0
Dimitrijević et al. 2014 [22]	2	0		
Dos Santos et al. 2023 [23]	1	0		

Dulla et al. 2024 [24]	1	0		
Freitas, & Massuça 2025 [25]	2	0		
Freitas, Ramos & Massuça 2025 [26]	3	1		
Heredia et al. 2024 [27]	1	0		
Hernandez et al. 2021 [28]	1	0		
Hernandez et al. 2021 [29]	2	0		
Iorga et al. 2024 [30]	1	0		
Janković 2023 [31]	1	0	1	0
Janković et al. 2015 [32]			3	1
Janković & Koropanovski 2024 [33]			1	0
Koropanovski et al. 2020 [34]	1	0		
Koropanovski et al. 2022 [35]	1	0		
Korre et al. 2019 [36]	1	0		
Kukić et al. 2023 [37]	3	0		
Kukić, Janković & Koropanovski 2023 [38]	2	0		
Kukić et al. 2019 [39]	1	0		
Kukić et al. 2019 [40]	1	0		
Kukić et al. 2020 [41]	2	0		
Kukić et al. 2022 [42]	1	1		
Kukić et al. 2024 [43]	2	0		
Lagestad & van den Tillaar 2014 [44]	1	0		
Lockie et al. 2023 [45]	2	0	1	0
Lockie 2024 [46]	2	0		
Lockie 2025 [47]	2	0		
Lockie et al. 2019 [48]	2	0		
Lockie et al. 2022 [49]			2	0

Lockie et al. 2021 [50]	2	0		
Lockie et al. 2018 [51]	1	0	1	0
Lockie et al. 2020 [52]	2	0		
Lockie et al. 2020 [53]	1	0		
Lockie et al. 2021 [54]	1	0		
Lockie et al. 2020 [55]	2	0		
Lockie et al. 2020 [56]	2	0		
Lockie et al. 2021 [57]	1	0		
Lockie et al. 2022 [58]			2	0
Lockie et al. 2018 [59]	2	0		
Lockie et al. 2020 [60]	1	0		
Lockie et al. 2020 [61]	1	0	1	0
Lockie et al. 2021 [62]	1	0	1	0
Lockie et al. 2021 [63]	2	0		
Lockie et al. 2024 [64]	1	0	1	0
Lockie et al. 2024 [65]	1	0	1	0
Lockie et al. 2020 [66]	2	0		
Lockie et al. 2025 [67]	2	0	1	0
Martinez & Abel 2021 [68]	2	0		
Martinez et al. 2022 [69]	2	0	1	0
Massuça et al. 2023 [70]	1	0		
Maupin et al. 2024 [71]	1	0	1	0
Maupin et al. 2020 [72]	1	0	1	0
Merrigan et al. 2022 [73]	1	0		
Moreno et al. 2019 [74]	1	0	1	0
Murphy et al. 2024 [75]	2	0	1	0

Orr et al. 2016 [76]	2	0		
Orr et al. 2017 [77]	1	0	1	0
Orr et al. 2021 [78]	1	0		
Orr et al. 2018 [79]	1	0		
Orr et al. 2020 [80]	2	0		
Orr, Ford & Stierli 2016 [81]	1	0		
Papadakis et al. 2021 [82]	1	0		
Poirier et al. 2022 [83]	1	1		
Poirier et al. 2023 [84]	1	0	1	0
Schram et al. 2024 [85]	1	0		
Shusko et al. 2017 [86]	1	0		
Sousa-Sá et al. 2023 [87]	1	0		
Sutton et al. 2025 [88]	1	0	1	0
Tomes et al. 2020 [89]	1	0		
Wu et al. 2015 [90]	1	0		
Koropanovski et al. 2022 [91]	2	0		
Lockie et al. 2022 [92]			2	0
Koedijk et al. 2023 [93]	1	0	1	0
Henze et al. 2024 [94]	1	0	2	0
Kolarević et al. 2014 [95]	1	0		
Krugly et al. 2023 [96]	2	0		
Poirier et al. 2021 [97]	1	0	1	0
Canetti et al. 2025 [98]	3	0		
Němá et al. 2025 [99]	1	0		
Jia 2025 [100]	3	0		
Janković & Dopsaj 2022 [101]	1	0		

References

1. Campbell, P.; Maupin, D.; Lockie, R.G.; Dawes, J.J.; Simas, V.; Canetti, E.; Schram, B.; Orr, R. The Development of Normative Fitness Data and Analyzing the Relationships Between 20MSFT and 2.4-km Run Performance in Australian Police Recruits. *J. Strength Cond. Res.* **2022**, *38*, 1951-1958.
2. Gonzales, S.M.; Orr, R.M.; Coburn, J.W.; Hoffmann, M.D.; Kennedy, K.; Dawes, J.J.; Lockie, R.G. A Retrospective Analysis of Southeastern US Police Recruit Health and Fitness From 4 Points in Time Within a 16-Year Period: Implications for Physical Fitness Training. *J. Strength Cond. Res.* **2024**, *38*, 1118-1126.
3. Marks, D.; Merrigan, J.J.; Martin, J. Do baseline physical fitness measures predict law enforcement academy graduation? *Work.* **2022**, *72*, 263-269.
4. Jeknić, V.; Stojković, M.; Bacetić, N. Fitness level comparison between police college freshman and senior students. *Int. J. Phys. Educ. Sports Health* **2018**, *5*, 99-104.
5. Annell, S.; Lindfors, P.; Sverke, M. Police selection–implications during training and early career. *Policing: Int. J. Police Srat. Manag.* **2015**, *38*, 221-238.
6. Jankovsky, P.; Sedlacek, J.; Balint, G. The level comparision of somatic parameters and general physical fitness of Bratislava Police Academy male students. *Gymnasium.* **2013**, *14*.
7. Ramos, N.; Massuça, L.M. Reliability of Police Physical Tasks and Fitness Predictors. *Appl. Sci.* **2025**, *15*, 9271.
8. Stojković, M.; Kukić, F.; Nedeljković, A.; Orr, R.M.; Dawes, J.J.; Čvorović, A.; Jeknić, V. Effects of a physical training programme on anthropometric and fitness measures in obese and overweight police trainees and officers. *S. Afr. Res. Sport Phys. Educ. Recreation.* **2021**, *43*, 63-76.
9. Thielgen, M.M.; Schade, S. Physical Job Demands in Police Officer Selection and Training: Normative Data and Predictive Validity—A Research Note. *J. Police Crim. Psychol.* **2024**, *39*, 660-670.
10. Bloodgood, A.M.; Dawes, J.J.; Orr, R.M.; Stierli, M.; Cesario, K.A.; Moreno, M.R.; Dulla, J.M.; Lockie, R.G. Effects of sex and age on physical testing performance for law enforcement agency candidates: Implications for academy training. *J. Strength Cond. Res.* **2021**, *35*, 2629-2635.
11. Bloodgood, A.M.; Moreno, M.R.; Cesario, K.A.; McGuire, M.B.; Lockie, R.G. An investigation of seasonal variations in the fitness test performance of law enforcement recruits. *Facta Universitatis, Series: Physical Education and Sport.* **2020**, 271-282.
12. Campbell, P.; Maupin, D.; Lockie, R.G.; Dawes, J.J.; Simas, V.; Canetti, E.; Schram, B.; Orr, R. Determining the Changes in Law Enforcement Recruit Aerobic Fitness Using the 2.4-km Run and 20-m Multistage Fitness Test. Does the Type of Test Matter? *J. Strength Cond. Res.* **2024**, *38*, 1111-1117.
13. Campbell, P.; Maupin, D.; Lockie, R.G.; Dawes, J.J.; Simas, V.; Canetti, E.; Schram, B.; Orr, R. Evaluating the variability between 20-m multistage fitness test estimating equations in law enforcement recruits. *J. Strength Cond. Res.* **2024**, *38*, 742-748.
14. Cervantes-Sanabria, J.; Camacho-Sánchez, G.; Hernández-Elizondo, J.; Carazo-Vargas, P.; Brazo-Sayavera, F.J.; Castillo-Hernández, I.M.; Salicetti-Fonseca, A. Age, sex and adiposity correlates of physical fitness in Costa Rican police academy cadets. *Pensar en Movimiento: Revista de ciencias del ejercicio y la salud.* **2025**, *23*, 72-94.

15. Cesario, K.; Dulla, J.; Blood Good, A.; Moreno, M.R.; Dawes, J.J.; Lockie, R.G. Relationships between assessments in a physical ability test for law enforcement: Is there redundancy in certain assessments? *Iin. J. Exerc. Sci.* **2018**, *11*, 1063-1073.
16. Cocke, C.; Dawes, J.; Orr, R.M. The use of 2 conditioning programs and the fitness characteristics of police academy cadets. *J. Athl. Train.* **2016**, *51*, 887-896.
17. Collins, K.S.; Christensen, B.K.; Orr, R.M.; Dulla, J.M.; Dawes, J.J.; Lockie, R.G. Analysis of total and segmental body composition relative to fitness performance measures in law enforcement recruits. *Int. J. Exerc. Sci.* **2022**, *15*, 245.
18. Crawley, A.A.; Sherman, R.A.; Crawley, W.R.; Cosio-Lima, L.M. Physical fitness of police academy cadets: Baseline characteristics and changes during a 16-week academy. *J. Strength Cond. Res.* **2016**, *30*, 1416-1424.
19. Čvorović, A.; Kukić, F.; Orr, R.M.; Dawes, J.J.; Jeknić, V.; Stojković, M. Impact of a 12-week postgraduate training course on the body composition and physical abilities of police trainees. *J. Strength Cond. Res.* **2021**, *35*, 826-832.
20. Dawes, J.; Lockie, R.G.; Orr, R.M.; Kornhauser, C.; Holmes, R. Initial fitness testing scores as a predictor of police academy graduation. *J. Aust. Strength Cond.* **2019**, *27*, 30-37.
21. Dawes, J.J.; Scott, J.; Canetti, E.F.; Lockie, R.G.; Schram, B.; Orr, R.M. Profiling the New Zealand police trainee physical competency test. *Front. Public Health.* **2022**, *10*, 821451.
22. Dimitrijević, R.; Koropanovski, N.; Dopsaj, M.; Vučković, G.; Janković, R. The influence of different physical education programs on police students' physical abilities. *Policing: Int. J. Police Strateg. Manag.* **2014**, *37*, 794-808.
23. Dos Santos, M.L.; Thompson, M.; Dinyer-McNeely, T.; Torrence, T.; Lockie, R.G.; Orr, R.M.; Dawes, J.J. Differences and relationships between push-up and sit-up variations among male law enforcement cadets. *J. Strength Cond. Res.* **2023**, *37*, 1865-1869.
24. Dulla, J.; Lockie, R.; Orr, R.; Schram, B.; Canetti, E. A profile of two different applicant test batteries with regards to sex and age in successful law enforcement applicants. *Work.* **2024**, *77*, 1359-1368.
25. Freitas, J.D.; Massuça, L.M. Fitness Profile of Police Officers from Rapid Intervention Teams of the Lisbon Metropolitan Command. *J. Funct. Morphol. Kinesiol.* **2025**, *10*, 90.
26. Freitas, J.D.; Ramos, S.A.; Massuça, L.M. Reliability of Critical Fitness Tests for Police Officers. *Appl. Sci.* **2025**, *15*, 405.
27. Heredia, C.E.; Dawes, J.J.; Dulla, J.M.; Orr, R.M.; Lockie, R.G. Shoulder Taps: Relationships Between a New Movement Screening Assessment with Body Composition and Physical Fitness in Law Enforcement Recruits. *Int. J. Exerc. Sci.* **2024**, *17*, 702.
28. Hernandez, E.; Dawes, J.J.; Dulla, J.; Orr, R.; Lockie, R.G. Fitness percentile rank data for female law enforcement recruits in the USA. *Facta Universitatis, Series: Physical Education and Sport.* **2021**, 109-118.
29. Hernandez, E.; Dawes, J.J.; Orr, R.M.; Dulla, J.M.; Lockie, R.G. Are there differences in fitness between recruits from larger (hosting) and smaller (participating) law enforcement agencies? *Int. J. Sci.* **2021**, *14*, 885.
30. Iorga, V.; De Hillerin, P.-J.; Dancescu, D.; Geanta, V.A. Importance of integrating psychophysical training into the holistic training program for law enforcement. *J. Phys. Educ. Sport.* **2024**, *24*, 1434-1444.
31. Janković, R. Correlation of basic and specific physical abilities: a study on the obstacle course for assessing specific agility of police officers. *SportLogia* **2023**, *19*.

32. Janković, R.Z.; Dopsaj, M.; Dimitrijević, R.; Savković, M.; Koropanovski, N.; Vučković, G. Validity and reliability of the test for assessment of specific physical abilities of police officers in the anaerobic-lactate work regime. *Facta Universitatis, Series: Physical Education and Sport.* **2015**, 19-32.
33. Janković, R.; Koropanovski, N. Correlation of Morphological Characteristics with Police Specific Fitness Test in Police Students. *SportLogia.* **2024**, *20*.
34. Koropanovski, N.; Kukić, F.; Janković, R.; Dimitrijević, R.; Dawes, J.J.; Lockie, R.G.; Dopsaj, M. Impact of physical fitness on recruitment and its association to study outcomes of police students. *S. Afr. J. Res. Sport Phys. Educ. Recreation.* **2020**, *42*, 23-34.
35. Koropanovski, N.; Kukić, F.; Janković, R.; Kolarević, D.; Subošić, D.; Orr, R.M. Intellectual potential, personality traits, and physical fitness at recruitment: Relationship with academic success in police studies. *Sage Open.* **2022**, *12*, 21582440221079932.
36. Korre, M.; Loh, K.; Eshleman, E.J.; Lessa, F.S.; Porto, L.; Christophi, C.A.; Kales, S.N. Recruit fitness and police academy performance: a prospective validation study. *Occup. Med.* **2019**, *69*, 541-548.
37. Kukić, F.; Janković, R.; Dawes, J.J.; Orr, R.; Koropanovski, N. Effects of occupational load on the acceleration, change of direction speed, and anaerobic power of police officers. *J. Strength Cond. Res.* **2023**, *37*, 1237-1243.
38. Kukić, F.; Janković, R.; Koropanovski, N. Association of physical abilities to academic success in police students. *SportLogia.* **2023**, *19*.
39. Kukic, F.; Jeknic, V.; Dawes, J.J.; Orr, R.M.; Stojkovic, M.; Cvorovic, A. Effects of training and a semester break on physical fitness of police trainees. *Kinesiology.* **2019**, *51*, 161-169.
40. Kukić, F.; Koropanovski, N.; Janković, R.; Dopsaj, M. Effects of specialized physical education and additional aerobic training on aerobic endurance of police students. *Hum. Sport. Med.* **2019**, *19*, 58-64.
41. Kukić, F.; Lockie, R.G.; Vesković, A.; Petrović, N.; Subošić, D.; Spasić, D.; Paspalj, D.; Vulin, L.; Koropanovski, N. Perceived and measured physical fitness of police students. *Int. J. Environ. Res. Public Health.* **2020**, *17*, 7628.
42. Kukić, F.; Orr, R.; Marković, M.; Dawes, J.J.; Čvorović, A.; Koropanovski, N. Factorial and Construct Validity of Sit-Up Test of Different Durations to Assess Muscular Endurance of Police Students. *Sustainability.* **2022**, *14*, 13630.
43. Kukić, F.; Zlojutro, N.; Paspalj, D.; Bajić, S.; Kovačević, S.; Vulin, L.; Ranđević, N.; Koropanovski, N. Effects of specific RAMP protocol phase on change of direction speed of police students. *J. Func. Morphol. Kinesiol.* **2024**, *9*, 194.
44. Lagestad, P.; Van den Tillaar, R. A comparison of training and physical performance of police students at the start and the end of three-year police education. *J. Strength Cond. Res.* **2014**, *28*, 1394-1400.
45. Lockie, R.; Dawes, J.J.; Sakura, T.; Schram, B.; Orr, R.M. Relationships between physical fitness assessment measures and a workplace task-specific physical assessment among police officers: A retrospective cohort study. *J. Strength Cond. Res.* **2023**, *37*, 678-683.
46. Lockie, R.G. 20-m Multistage Fitness Test Normative Percentile Rank Data for Law Enforcement Recruits. *Sportlogia.* **2024,** *21*, 12-23.
47. Lockie, R.G. Percentile Rank Data for the Countermovement Vertical Jump Measured by a Jump-and-Reach Device in Law Enforcement Recruits. *SportLogia.* **2025**, *21*.
48. Lockie, R.G.; Balfany, K.; Bloodgood, A.M.; Moreno, M.R.; Cesario, K.A.; Dulla, J.M.; Dawes, J.J.; Orr, R.M. The influence of physical fitness on reasons for academy separation in law enforcement recruits. *Int. J. Environ. Res. Public. Health.* **2019**, *16*, 372.

49. Lockie, R.G.; Beitzel, M.M.; Dulla, J.M.; Dawes, J.J.; Orr, R.M.; Hernandez, J.A. Between-sex differences in the Work Sample Test Battery performed by law enforcement recruits: Implications for training and potential job performance. *J. Strength Cond. Res.* **2022**, *36*, 1310-1317.
50. Lockie, R.G.; Carlock, B.N.; Ruvalcaba, T.J.; Dulla, J.M.; Orr, R.M.; Dawes, J.J.; McGuire, M.B. Skeletal muscle mass and fat mass relationships with physical fitness test performance in law enforcement recruits before academy. *J. Strength Cond. Res.* **2021**, *35*, 1287-1295.
51. Lockie, R.G.; Dawes, J.J.; Balfany, K.; Gonzales, C.E.; Beitzel, M.M.; Dulla, J.M.; Orr, R.M. Physical fitness characteristics that relate to Work Sample Test Battery performance in law enforcement recruits. *Int. J. Environ. Res. Public Health.* **2018**, *15*, 2477.
52. Lockie, R.G.; Dawes, J.J.; Dulla, J.M.; Orr, R.M.; Hernandez, E. Physical fitness, sex considerations, and academy graduation for law enforcement recruits. *J. Strength Cond. Res.* **2020**, *34*, 3356-3363.
53. Lockie, R.G.; Dawes, J.J.; D MACLEAN, N.; Pope, R.P.; Holmes, R.J.; Kornhauser, C.L.; Orr, R.M. The impact of formal strength and conditioning on the fitness of law enforcement recruits: A retrospective cohort study. *Int. J. Exerc. Sci.* **2020**, *13*, 1615.
54. Lockie, R.G.; Dawes, J.J.; Moreno, M.R.; Cesario, K.A.; Balfany, K.; Stierli, M.; Dulla, J.M.; Orr, R.M. Relationship between the 20-m multistage fitness test and 2.4-km run in law enforcement recruits. *J. Strength Cond. Res.* **2021**, *35*, 2756-2761.
55. Lockie, R.G.; Dawes, J.J.; Moreno, M.R.; McGuire, M.B.; Ruvalcaba, T.J.; Bloodgood, A.M.; Dulla, J.M.; Orr, R.M. We need you: Influence of hiring demand and modified applicant testing on the physical fitness of law enforcement recruits. *Int. J. Res. Public Health.* **2020**, *17*, 7512.
56. Lockie, R.G.; Dawes, J.J.; Orr, R.M.; Dulla, J.M. Recruit fitness standards from a large law enforcement agency: Between-class comparisons, percentile rankings, and implications for physical training. *J. Strength Cond. Res.* **2020**, *34*, 934-941.
57. Lockie, R.G.; Dawes, J.J.; Orr, R.M.; Dulla, J.M. Physical fitness: Differences between initial hiring to academy in law enforcement recruits who graduate or separate from academy. *Work.* **2021**, *68*, 1081-1090.
58. Lockie, R.G.; Dawes, J.J.; Orr, R.M.; Dulla, J.M. The bigger they are: Relationships between body height and mass with the body drag task in law enforcement recruits. *Int. J. Exerc, Sci.* **2022**, *15*, 570.
59. Lockie, R.G.; Dawes, J.J.; Orr, R.M.; Stierli, M.; Dulla, J.M.; Orjalo, A.J. Analysis of the effects of sex and age on upper-and lower-body power for law enforcement agency recruits before academy training. *J. Strength Cond. Res.* **2018**, *32*, 1968-1974.
60. Lockie, R.G.; Hernandez, J.A.; Moreno, M.R.; Dulla, J.M.; Dawes, J.J.; Orr, R.M. 2.4-km run and 20-m multistage fitness test relationships in law enforcement recruits after academy training. *J. Strength Cond. Res.* **2020**, *34*, 942-945.
61. Lockie, R.G.; Moreno, M.R.; McGuire, M.B.; Ruvalcaba, T.J.; Bloodgood, A.M.; Dulla, J.M.; Orr, R.M.; Dawes, J.J. Relationships between isometric strength and the 74.84-kg (165-lb) body drag test in law enforcement recruits. *J. Hum. Kinet.* **2020**, *74*, 5.
62. Lockie, R.G.; Moreno, M.R.; Rodas, K.A.; Dulla, J.M.; Orr, R.M.; Dawes, J.J. With great power comes great ability: Extending research on fitness characteristics that influence Work Sample Test Battery performance in law enforcement recruits. *Work.* **2021**, *68*, 1069-1080.
63. Lockie, R.G.; Orr, R.M.; Dulla, J.; Dawes, J.J. Relationships between Absolute and Relative Lower-Body Power and Foot Pursuit Ability in Law Enforcement Recruits. *Int. J. Exerc. Sci.* **2021**, *14*, 1078-1089.
64. Lockie, R.G.; Orr, R.M.; Kennedy, K.; Dawes, J.J. Introduction of an applicant Job-Related Task Assessment (JTA) and the effects on the health and fitness of police recruits. *Work.* **2024**, *77*, 1401-1413.
65. Lockie, R.G.; Orr, R.M.; Sanchez, K.J.; Gonzales, S.M.; Viramontes, E.; Kennedy, K.; Dawes, J.J. Relationships between physical ability test performance and fitness in recruits from a Southeastern US police department. *J. Strength Cond. Res.* **2024**, *38*, 1472-1478.

66. Lockie, R.G.; Ruvalcaba, T.R.; Stierli, M.; Dulla, J.M.; Dawes, J.J.; Orr, R.M. Waist circumference and waist-to-hip ratio in law enforcement agency recruits: Relationship to performance in physical fitness tests. *J. Strength Cond. Res.* **2020**, *34*, 1666-1675.
67. Lockie, R.G.; Young, M.A.; Lanham, S.N.; Orr, R.M.; Dawes, J.J.; Nagel, T.R. Retrospective Analysis of Grip and Index Finger Strength, Job-Specific Scenario, and Shooting Performance in Incumbent Law Enforcement Personnel and Cadets. *J. Strength Cond. Res.* **2025**, *39*, e581-e588.
68. Martinez, G.J.; Abel, M.G. Effect of a law enforcement academy training program on validated fitness outcomes of cadets. *J. Strength Cond. Res.* **2021**, *35*, 955-962.
69. Martinez, G.J.; Ma, X.; Best, S.; Johnson, B.F.; Abel, M.G. Implementation of High Intensity Interval Training and Autoregulatory Progressive Resistance Exercise in a Law Enforcement Training Academy. *Int. J. Exerc. Sci.* **2022**, *15*, 1246.
70. Massuça, L.M.; Monteiro, L.; Coutinho, G.; Santos, V. Four-year training course for police officers (CFOP) and fitness outcomes of police academy cadets: A cohort study from 2004 to 2020. *Healthcare.* 2023; p. 2901.
71. Maupin, D.; Canetti, E.F.; Schram, B.; Dulla, J.M.; Lockie, R.G.; Dawes, J.J.; Orr, R.M. Law enforcement recruit fitness: Changes across the fitness spectrum. *Work.* **2024**, *79*, 947-963.
72. Maupin, D.; Schram, B.; Canetti, E.; Dawes, J.; Lockie, R.; Orr, R. Developing the Fitness of Law Enforcement Recruits during Academy Training. *Sustainability.* **2022**, *12*, 7944.
73. Merrigan, J.J.; Burke, A.A.; Eddo, O.O.; Kearney, J.; Marks, D.; Martin, J.R. Upper body push to pull ratios in law enforcement officer recruits. *Work.* **2022**, *73*, 1167-1174.
74. Moreno, M.R.; Dulla, J.; Dawes, J.; Orr, R.M.; Cesario, K.A.; Lockie, R.G. Lower-body power and its relationship with body drag velocity in law enforcement recruits. *Int. J. Exerc. Sci.* **2019**, *12*, 847-858.
75. Murphy, M.C.; Merrick, N.; Mosler, A.B.; Allen, G.; Chivers, P.; Hart, N.H. Cardiorespiratory fitness is a risk factor for lower-limb and back injury in law enforcement officers commencing their basic training: a prospective cohort study. *Res. Sports. Med.* **2024**, *32*, 511-523.
76. Orr, R.; Pope, R.; Peterson, S.; Hinton, B.; Stierli, M. Leg power as an indicator of risk of injury or illness in police recruits. *Int. J. Environ. Res. Public Health.* **2016,** *13*, 237.
77. Orr, R.; Pope, R.; Stierli, M.; Hinton, B. Grip strength and its relationship to police recruit task performance and injury risk: A retrospective cohort study. *Int. J. Environ. Res. Public Health.* **2017**, *14*, 941.
78. Orr, R.M.; Canetti, E.; Movshovich, J.; Lockie, R.; Dawes, J.; Schram, B. Profiling the New Zealand police physical appraisal test. *Int. J. Emerg. Serv.* **2021**, *10*, 266-275.
79. Orr, R.M.; Dawes, J.J.; Pope, R.; Terry, J. Assessing differences in anthropometric and fitness characteristics between police academy cadets and incumbent officers. *J. Strength Cond. Res.* **2018**, *32*, 2632-2641.
80. Orr, R.M.; Ferguson, D.; Schram, B.; Dawes, J.J.; Lockie, R.; Pope, R. The relationship between aerobic test performance and injuries in police recruits. *J. Exerc. Sci.* **2020**, *13*, 1052.
81. Orr, R.M.; Ford, K.; Stierli, M. Implementation of an ability-based training program in police force recruits. *J. Strength Cond. Res.* **2016**, *30*, 2781-2787.
82. Papadakis, Z.; Stamatis, A.; Kukic, F.; Koropanovski, N. Moving past the one-size-fits-all education-training model of police academies to the self-prescribed individualized exercise prescription model. *Int. J. Environ. Res. Public Health.* **2021**, *18*, 11676.

83. Poirier, S.; Gendron, A.; Trudeau, F.; Lajoie, C. Cardiorespiratory fitness in police recruits: Assessing the validity of the 20-meter shuttle run test for recruitment purposes. *Work.* **2022**, *71*, 1193-1201.
84. Poirier, S.; Houle, J.; Lajoie, C.; Trudeau, F. Cardiorespiratory fitness of police recruits: Normative reference values and temporal trend. *J. Strength Cond. Res.* **2023**, *37*, 207-212.
85. Schram, B.; Kukić, F.; Janković, R.; Dimitrijević, R.; Žigić, G.; Orr, R.; Koropanovski, N. Effects of a single-day pre-academy physical test training session on physical fitness scores of police candidates. *Work.* **2024**, *77*, 1369-1375.
86. Shusko, M.; Benedetti, L.; Korre, M.; Eshleman, E.J.; Farioli, A.; Christophi, C.A.; Kales, S.N. Recruit fitness as a predictor of police academy graduation. *Occup. Med.* **2017**, *67*, 555-561.
87. Sousa-Sá, E.; Pereira, S.; Júdice, P.B.; Monteiro, L.; Massuça, L.M. COVID-19 Social Restrictions' Impact on the Health-Related Physical Fitness of the Police Cadets. *Healthcare.* **2023**; p. 1949.
88. Sutton, V.R.; Murphy, M.C.; McCaskie, C.J.; Chivers, P.T.; Hart, N.H.; Cochrane Wilkie, J.L.; Allen, G.; Dalla Via, J. The Association of Body Composition and Musculoskeletal Characteristics with Police Recruit Performance: A Cross-Sectional Study. *J. Func. Morphol. Kinesiol.* **2025**, *10*, 132.
89. Tomes, C.; Schram, B.; Pope, R.; Orr, R. What is the impact of fitness on injury risk during police academy training? A retrospective cohort study. *BMC sports Sci. Med. Rehabil.* **2020**, *12*, 39.
90. Wu, Y.-N.; Hallbourg, K.W.; Collins, S.M. Changes of general fitness and muscle properties following police cadet training. *J. Phys. Ther. Sci.* **2015**, *27*, 2783-2786.
91. Koropanovski, N.; Orr, R.M.; Dopsaj, M.; Heinrich, K.M.; Dawes, J.J.; Kukic, F. Effects of maximal and submaximal anaerobic and aerobic running on subsequent change-of-direction speed performance among police students. *Biology.* **2022**, *11*, 767.
92. Lockie, R.G.; Moreno, M.R.; Dawes, J.J.; Orr, R.M.; Rodas, K.A.; Dulla, J.M. An analysis of the body drag test in law enforcement recruits with consideration to current population demographics. *Int. J. Exerc. Sci.* **2022**, *15*, 276.
93. Koedijk, M.; Hutter, R.; Renden, P.G.; Kleygrewe, L.; Oudejans, R.R. Fitness testing at police academies: Optimal fitness for duty. *Int. J. Police Sci. Manag.* **2023**, *25*, 170-182.
94. Henze, K.M.; Klausen, L.K.; Martín, G.F.; Dillern, T. Physical Fitness and Use-of-Force Performance for Police Students: The Impact of Body Height, Body Mass, Age, and Gender. *Nordic Journal of Studies in Policing* **2024**, *11*, 1-23.
95. Kolarevic, D.; Dimitrijevic, R.; Vuckovic, G.; Koropanovski, N.; Dopsaj, M. Relations between psychological characteristics and physical abilities in a sample of female police candidates. *Open Sports Sci. J.* **2014**. *7*, 22-28.
96. Krugly, S.; Bjärsholm, D.; Jansson, A.; Rosendal Hansen, A.; Hansson, O.; Brehm, K.; Datmo, A.; Hafsteinsson Östenberg, A.; Vikman, J. A retrospective study of physical fitness and mental health among police students in Sweden. *Police J.* **2023**, *96*, 430-450.
97. Poirier, S.; Gendron, A.; Gendron, P.; Lajoie, C. Fitness components associated with performance of a law enforcement physical employment standard in police cadets. *J. Sports Med. Phys. Fitness.* **2021**, *62*, 981-989.
98. Canetti, E.F.D.; Schram, B.; Pope, R.; Lockie, R.G.; Dawes, J.J.; Campbell, P.; Orr, R. Test-Retest Reliability of the 20-Metre Shuttle Run Test in a Cohort of Police Trainees. *SportLogia.* **2025**, *21*, 1-7, doi:10.63356/spl.2025.005.

99. Němá, K.; Kozák, T.; Ligday, M.; Rydzik, Ł. Sport Mont 23 (2025) 3: 13-18 13 Physical Fitness of Police Force Applicants: Trends from 2010 to 2023. *Sport Mont.* **2025**, *23*, 13-18, doi:10.26773/smj.251002.
100. Jia, D. Graduation-stage physical fitness as a predictor of operational performance and injury risk among police recruits: a prospective cohort study. *BMJ Open Sport Exerc. Med.* **2025**, *11*, e002871, doi:10.1136/bmjsem-2025-002871.
101. Jankovic, R.; Dopsaj, M. Sprint running, agility and anaerobic endurance: Standards for the students at the University of Criminal Investigation and Police Studies. *Nauka bezbednost policija.* **2022**, *27*, 45-58, doi:10.5937/nabepo27-36046.
